# A decade of discourse: Exploring sentiments and trends around immigration on social media from 2014 to 2024

**DOI:** 10.1016/j.socscimed.2025.118715

**Published:** 2025-10-25

**Authors:** Thu T. Nguyen, Penchala Sai Priya Mullaputi, Xiaohe Yue, Heran Mane, Adwaith Santhosh, Elizabeth Dennard, Amrutha S. Alibilli, Katrina Makres, Rozalina G. McCoy, Quynh C. Nguyen

**Affiliations:** a Department of Epidemiology and Biostatistics, University of Maryland, College Park, College Park, MD, United States; b Department of Data Science, College of Computer, Mathematical, and Natural Sciences, University of Maryland, College Park, College Park, MD, United States; c Division of Gerontology, Department of Epidemiology and Public Health, University of Maryland School of Medicine, Baltimore, MD, United States; d University of Maryland Institute for Health Computing, North Bethesda, MD, United States; e Division of Endocrinology, Diabetes, and Nutrition, Department of Medicine, University of Maryland School of Medicine, Baltimore, MD, United States

**Keywords:** Social media, Refugees, Immigrants, User engagement, Sentiment analysis

## Abstract

**Introduction:**

Social media discussions contribute to the evolving public perception of refugees and immigrants. However, prior research often relied on a single platform and short-term analyses, offering a fragmented view of a highly dynamic phenomenon.

**Objective:**

Examine trends in public narratives surrounding refugees and immigrants, including the evolution of sentiment and user engagement on Twitter, Facebook, and Bluesky.

**Methods:**

We analyzed 6.3 million U.S.-based English-language posts from Twitter (2014–2023), Facebook (2014–2024), and Bluesky (2023–2024), using platform APIs. Posts containing one or more of 129 immigration-related keywords were grouped into 76 categories. Sentiment was classified using a supervised Support Vector Machine model, and engagement was aggregated at the keyword level. Twitter geodata enabled state-level sentiment mapping.

**Results:**

Peaks in volume and negativity aligned with major events, including the 2014 Syrian refugee crisis, the 2017 travel ban, and the 2018 family separation policy. From 2014 to 2019, negative sentiment increased on both Twitter and Facebook, then became more neutral in subsequent years. Bluesky began with predominantly neutral discourse in 2023 but grew more negative after its public launch. Refugee-related discourse was consistently less negative than immigrant-related discourse across all platforms, while enforcement-related and exclusionary rhetoric keywords emerged as the most negatively evaluated. Twitter geodata revealed widespread negativity across states, although refugee discourse remained more moderate or neutral than immigrant discourse overall.

**Conclusion:**

Migration discourse is shaped by political events, emotional framing, and platform-specific dynamics, underscoring the need for cross-platform analyses to understand evolving digital narratives.

## 1. Introduction

For millions of refugees and immigrants, migration is not a voluntary act but a necessity. Whether escaping war, persecution, or economic despair, they embark on uncertain journeys in search of safety, dignity, and a better future. In today’s digital era, their stories are not only reported through traditional media but are continuously shaped in real time through social media platforms like Twitter (now known as X), Facebook, and Bluesky. These platforms have become battlegrounds of public opinion, where narratives about refugees and immigrants oscillate between empathy, hostility, advocacy, and misinformation. Unlike curated news coverages, social media allows individuals to shape narratives in real time. Twitter hashtags, Facebook groups, and Bluesky discussions contribute to the evolving perception of refugees and immigrants, often reflecting concerns, anxieties, or political polarization surrounding migration (Inuwa-Dutse et al., 2020). Beyond their discursive role, digital platforms also provide unique advantages as research tools. Recent scholarship highlights the value of digital data collection. Ndiinee and Gever (2025) demonstrate that research participants often prefer online modes of engagement compared to face-to-face approaches, highlighting convenience and accessibility as key factors. In addition, Gever (2024) outlines several advantages of digital data collection over face-to-face methods, including the ability to gather large and diverse datasets, cost-effectiveness, and improved access to information. Together, these studies support the growing relevance of digital platforms, such as social media, as robust sources of data for examining migration discourse.

Migration discourse has been historically studied through an economic or sociological lens, but there has been a recent shift towards interdisciplinary approaches and integration of multimodal data sources. For instance, Goodman and Omidiora (2025) investigated Nigerian films to gain insights into Nigerian youth’s emigration motivations and experiences. Just as films have been analyzed to capture public sentiment, attitudes, and behaviors surrounding migration, social media represents another untapped data source for identifying migration narrative trends for diverse demographic populations.

In comparison to traditional survey methodologies, social media platforms offer less regulated environments for public discourse, often functioning as echo chambers that amplify stereotypes and prejudices (Swastiningsih et al., 2024). Due to its perceived anonymity, it fosters disinhibition, allowing users to express views they might not share offline (Zhao, 2024). Furthermore, social media misinformation creates significant risk for migrant populations. For example, 70 % of the U. S.-bound Central American migrants reported relying on Facebook for migration-related information, heightening risks of fraud, and exploitation (Tech Transparency Project, 2022). Therefore, social media offers a valuable data source for investigating perceptions of solidarity, hostility, and misinformation for discussions surrounding refugees and immigrants.

### 1.1. The role of social media in shaping anti-immigrant sentiment

A growing body of research has explored the role of social media in shaping public perceptions of refugees and immigrants. Researchers have employed a range of advanced analytical methods including machine learning models that have enabled large-scale analyses of online data. Social media produces a large volume of textual and image data, making text mining, natural language processing, and multimodal models (models that can analyze multiple types of data simultaneously) indispensable for understanding conversations, tracking emerging topics, and evaluating public sentiment. For instance, Gruzd et al. (2024) analyzed anti-refugee discourse on Twitter during the 2022 Russian invasion of Ukraine using the Google Perspective API, finding 33 % of posts referenced partisan content and 53 % aligned with pro-Kremlin narratives, indicating how social media can contribute to polarized views on refugees. Similarly, Ahmed et al. (2024) integrated computational text analysis of immigration-related comments on Singapore Facebook pages with surveys. They found that online discussions often expressed negative sentiment and economic concerns with higher social media usage correlating with increased anti-immigrant sentiment. These studies highlight the importance of examining platform-specific patterns with advanced strategies like machine learning models to understand how immigration discourse circulates across different social media environments. Qualitative studies further emphasize the role of emotional language in anti-immigrant discourse. Gündoğmuş and Mete (2024) found that posts that frame immigrants as threats to national security or economic stability often tap into fear-based emotions and anti-immigrant narratives.

Further studies conducted on migration discourse highlight the importance of framing in shaping public perception. During the 2022 Russian invasion of Ukraine, European media narratives on Twitter shifted over time with sentiment increasingly portraying Ukrainian refugees in a favorable light (Wildemann et al., 2023). The terminology used in digital discussions, such as “refugee” versus “migrant,” significantly shapes public perception, influencing whether migrants are viewed through a humanitarian or economic lens (Lee and Nerghes, 2018). Mendelsohn et al. (2021) further emphasize that social media framing, user ideology, and regional context drive audience engagement with human interest, cultural, and political frames influencing the tone and perception of immigration discussions. Collectively, these findings illustrate the critical role of framing and sentiment in shaping digital migration discourse, demonstrating how linguistic and narrative strategies can shift public attitudes and engagement.

Framing dynamics extend beyond perception to produce tangible societal consequences. Polarizing social media rhetoric against ethnic and religious minorities has been linked to the instigation of violence (Wahlström et al., 2020). In Germany, Müller and Schwarz (2020) demonstrate that spikes in anti-refugee rhetoric on Facebook were followed by increases in hate crimes against refugees, highlighting how online hostility can spill into offline violence. In the United States, Hommadova-Lu and Mejova (2025) show that immigration discourse on Twitter shifts with electoral cycles; these fluctuations reinforce partisan divides, shape regional attitudes toward immigrants, and elevate the salience of immigration in public debate. At the same time, social media also serves as a platform for youth civic engagement around immigration, such as advocacy for the DREAM Act (Wilf et al., 2022). Taken together, these studies illustrate how immigration narratives on social media reverberate through broader society, influencing both public behavior and political discourse.

### 1.2. The gaps in existing research

Previous studies have leveraged social media to analyze users’ immigration attitudes and opinions. For instance, Grover et al. (2019) explored key events from November 2016 to 2017, including the Muslim Ban and DACA repeal, and found that pro-immigration tweets emphasized concerns related to preventing harm and promoting fairness, while anti-immigration tweets were associated with fear of subversion and negative sentiment. These previous studies highlight the influence of social media in shaping public perceptions of immigrants, shedding light upon the digital dynamics impacting minoritized populations.

Despite significant progress in this field, most existing studies heavily rely on short-term sentiment analysis, with limited exploration of long-term trends in migration discourse. This is partly because immigration sentiment often spikes around specific events (e.g., refugee crises, border policy changes, political elections), making short-term analyses more common. For instance, Sievers et al. (2021) explored migration-related data on Twitter during the COVID-19 pandemic, revealing shifts in sentiment and rising instances of discrimination across multiple countries. However, analyzing sentiment over longer periods can provide deeper insights into how migration attitudes evolve in response to demographic shifts, media influence, or political changes. A further limitation in the field is the overreliance on single-platform analysis, with much of the existing literature centered on Twitter, thereby excluding the perspectives that may be represented on other platforms. This narrow focus overlooks cross-platform differences in how migration discourse circulates. This study aims to bridge that gap by examining how public discourse surrounding refugees and immigrants evolves across Twitter, Facebook, and Bluesky from 2014 to 2024.

There are distinct differences in platform features and user demographics of Facebook, Twitter, and BlueSky. Facebook is one of the most widely used social media platforms worldwide with approximately 70 % of U.S. adults reporting using the platform and a high proportion of users identifying as older adults and women between the ages of 30 and 49 (Pew Research Center, 2024; Schaeffer, 2024). This usage also varies by race and ethnicity, with Black users reporting the highest usage at 73 %, followed by White (70 %), Hispanic (69 %), and Asian users (65 %). In contrast, 21 % of U.S. adults report using Twitter with a historical demographic trend towards younger, male users (Hughes and Wojcik, 2019). The share of U.S. adults who use Twitter is 19 % among White adults, 24 % among Black adults, 22 % among Hispanic adults, and 26 % among Asian adults. Additionally, about one third of Twitter posts from U.S. adults involve political content, underscoring the platform’s political reach (Bestvater, 2022).

Bluesky was first announced in 2019 as Twitter’s secondary venture before it became an independent company in 2021. By 2023, Bluesky became an invitation-only microblogging platform during its open beta testing phase and attracted over 1 million users. In February 2024, Bluesky announced its public launch and subsequently experienced a surge in new users (Marquit, 2025). While sharing various core features with its Twitter predecessor, Bluesky starkly differs in its open, decentralized architecture, which empowers users and allows them to customize their experience and content. Additionally, detailed demographic breakdowns on Bluesky remain sparse. However, Pew research suggests that the platform tends to be politically left-leaning (Stocking et al., 2025), while Facebook skews slightly more Republican, and Twitter remains more evenly split across party lines. While clear distinctions exist across platforms, social media users tend to maintain active accounts on several social media sites (Murdock et al., 2023). By studying behavior across platforms, the study aims to derive a more complete picture of online interactions, revealing what is unique to each platform and what is consistent across them.

By analyzing sentiment trends, keyword frequency, user engagement, and geographic distribution from a variety of platforms, the study explores both the emotional tone and visibility of refugee and immigration narratives. Specifically, it addresses questions such as: Which immigration-related keywords are most prevalent? How does sentiment evolve over time, and are there spikes during major immigration-related events? Which keywords spark more engagement, and how do these patterns vary across platforms? Do refugee-related discussions display similar or distinct sentiment patterns compared to immigrant-related discussions? How is sentiment for posts referencing refugees and immigrants distributed geographically across the United States? Through these questions, the study aims to gain a deeper understanding of long-term trends in migration discourse.

## 2. Methods

### 2.1. Social media data collection

For this observational study, data collection was restricted to publicly available, English-language posts from the U.S. for Twitter and Facebook and globally for Bluesky. All posts contained one or more refugees and immigrant-related keywords (see supplementary eTable 1). While Twitter and Facebook allow country-level filtering, Bluesky’s API does not, so its results are treated as supplemental. A keyword list of 129 terms was developed from prior studies and government glossaries (TRAC Immigration, 2023; USCIS, 2019; OHSS, 2024). To maximize coverage, the list included linguistic variations. To enhance analytical clarity, terms were grouped into 76 broader categories based on semantic similarity (e.g., “deport,” “deported” grouped under “deportation”). This grouping strategy minimized redundancy, standardized analysis across platforms, and enabled more effective identification of dominant themes. All social media posts meeting the inclusion criteria were included in the analysis. The analytic sample included 6,361,320 social media posts.

### 2.2. Twitter API

We collected and filtered data based on our immigration-related keywords, resulting in a data set with 1,956,170 tweets from January 2014 to March 2023. The data were collected using the Twitter Academic Research API, which has since been discontinued following the platform’s transition to X. We restricted the collection to US- based tweets in English with available location information. Metadata included tweet text, conversation ID, timestamp, and user-level attributes such as username, user ID, follower, and following counts.

The tweet location data is stored in a “place” object, which provides structured location information associated with the tweet including location ‘full_name’ along with the associated ‘place_type,’ which specifies the characteristic of the place. This place type can include categories such point of interest (POI) (e.g. ‘The White House’), neighborhood (e.g., ‘Upper West Side; Manhattan’), city (e.g., ‘Houston; TX’), admin (e.g., ‘Alaska; USA’), and country (e.g., ‘U.S.’). Additionally, the object includes a ‘bounding_box’ field, which defines the geographic area of the location as a polygon (X, 2019). Using these geographical fields, we standardized the data using rule-based parsing and reverse geocoding with Nominatim (Nominatim, 2025; OpenStreetMap, 2025). For POI and neighborhood location types, the bounding box midpoints were converted to coordinates; for city/admin names, geographic conventions were parsed directly. When metadata was missing, a user-reported location was used. Overall, 99.6 % (1,947,797 out of 1, 956,170) of tweets in the dataset were successfully geocoded, The remaining 0.4 % failed the geocoding process because of the ambiguous or incomplete place names and were excluded prior to geographic analyses.

### 2.3. Facebook

Leveraging Meta’s Content Library dashboard, 3,346,463 publicly available posts were collected from January 2014 to December 2024. The dataset included content from verified profiles, profiles with at least 25,000 followers and public pages with more than 15,000 followers or likes. To ensure relevance, we filtered for English-language posts where ‘*Post Surface Country,*’ indicating the country of the page administrator or profile owner, was listed as the U.S. Although the Meta Content Library includes data from platforms such as Instagram and Threads, these were excluded from our analysis due to the lack of geographic filtering capabilities for U.S.-based users.

### 2.4. Bluesky

As additional analyses, we collected 1,058,687 Bluesky posts using the platform’s public API. Data collection was restricted to English language posts between January 2023 and December 2024 due to the platform’s public launch in early 2023. The dataset included 120,504 posts from 2023 to 938,183 posts from 2024, reflecting substantial increases in the platform’s popularity over time. Metadata included user information (e.g., author ID, handle), post content (e.g., text, date, language), engagement metrics (e.g., likes, reposts), and multimedia information (e.g., image links, alt-text).

### 2.5. Standardized preprocessing pipeline

After collecting data from each platform, standard cleaning procedures were applied to ensure consistency across datasets from all platforms. These included the removal of duplicate entries, exclusion of posts missing key fields, and basic text preprocessing such as lower-casing, stopword removal, and lemmatization.

### 2.6. Sentiment analysis

Sentiment analysis used Support Vector Machines (SVMs), a supervised machine learning algorithm. SVMs require labeled training data to learn how to distinguish between positive and negative sentiment. Our SVM models were trained on data compiled from three publicly available sentiment-labeled datasets: Sentiment140 (n = 498), Kaggle (n = 7086), and Sanders (n = 5113), with an additional 6471 posts manually annotated by our research team. Model performance was evaluated using five-fold cross-validation. Accuracy is defined as the proportion of correctly classified tweets relative to the total number of tweets in the test data set. The F1 score, which combines precision (the proportion of predicted negatives that are correct) and recall (the proportion of actual negatives correctly identified), provides a balanced measure of performance. A higher F1 score reflects greater reliability in detecting tweets labeled as negative (1 = negative and 0 = not negative). The final model achieved an accuracy of 91 % and an F1 score of 84 % for negative sentiment classification, and 89 % accuracy with an F1 score of 81 % for positive sentiment classification. The final model was applied across Twitter, Facebook, and Bluesky datasets to classify sentiment in individual posts. The trained SVM model generated two binary outputs per post: one for positive sentiment and one for negative sentiment. A value of 1 indicated the presence of the sentiment, while 0 indicated its absence. Posts with both values set to 0 were labeled as “Neutral,” while posts with both values set to 1 were labeled as “Mixed.” Mixed posts represented less than 1 % of all data and were excluded from subsequent analyses to ensure clarity in interpretation.

### 2.7. Sentiment mapping

To analyze sentiment trends at the keyword group level, we calculated two metrics: an average sentiment score and a neutrality ratio. Each sentiment label was assigned a numeric value: positive = +1, negative = −1, and neutral = 0. For each keyword group, we computed a weighted average sentiment score by multiplying the count of posts in each sentiment category by its assigned value, summing these products, and dividing by the total number of sentiment-labeled tweets in that group. This yielded a single value representing the overall emotional tone associated with each keyword group.

In addition, we also computed a neutrality ratio, defined as the proportion of posts in each group that were labeled as neutral. This additional metric helped distinguish between genuinely neutral discussions and highly polarized ones in which positive and negative sentiment balanced out numerically. For instance, a sentiment score of zero may result either from neutral discussions or from a heated debate where half the tweets are positive and half are negative. In both cases, the average score is neutral, but the emotional dynamics are completely different. The neutrality ratio provided essential context for interpreting such cases.

### 2.8. Geographic variation in sentiment

To examine geographic variation in sentiment of posts referencing refugees and immigrants, we conducted sentiment mapping using data from Twitter (2014–2023), the only platform with available geolocation metadata. For overall sentiment maps, we calculated the average sentiment score per state across all immigration-related keywords. To generate separate maps for refugees and immigrants, we further grouped posts by both state and keyword type (i.e., “refugee” vs. “immigrant”) and computed average sentiment scores for each combination. The resulting aggregated sentiment scores were then visualized using Tableau, a data visualization software that supports geographic mapping and customizable color scales (Tableau, 2024).

### 2.9. Keyword frequency vs user engagement

We quantified users’ attention and interaction for each keyword group by calculating an engagement score per post, defined as the sum of user engagement statistics available for each platform.

**Table:** 

Platforms	User engagement statistics
Twitter	tweets retweet count, tweets reply count, tweets like count, tweets quote count
Facebook	angry count, care count, comment count, haha count, like count, love count, reaction count, sad count, share count, views, wow count
Bluesky	reply count, repost count, like count

Aggregating these scores at the keyword group level yielded two key metrics: the frequency of appearances and the total engagement generated. To evaluate keyword performance, we calculated engagement per keyword appearance by dividing the total engagement (sum of likes, retweets, and replies) by the number of times each keyword group appeared. This metric allowed us to identify topics that generated high engagement despite being mentioned less frequently, as well as those that were discussed often but generated low engagement. Median values of both keyword frequency and engagement per appearance were used to divide keyword groups into four distinct performance quadrants based on whether the frequency or engagement was above or below the median: low frequency, high engagement (overperformers), high frequency, low engagement (underperformers), high frequency, high engagement, and low frequency, low engagement. Each quadrant was visualized using a log-log scatter plot, and top- and bottom-performing keyword groups were annotated for interpretability. This quadrant-based approach offered a more nuanced understanding of keyword performance, enabling the identification of both highly discussed but low-impact topics, as well as niche topics with outsized audience responses.

### 2.10. Ethical considerations

This study was determined not to be human participant research by the University of Maryland College Park Institutional Review Board (2072551–1). The social media posts were anonymized, upholding user privacy.

## 3. Results

Overall, we analyzed 1,956,170 Twitter posts (2014–2023), 3,346,463 Facebook posts (2014–2024), and 1,058,687 Bluesky posts (2023–2024) containing at least one of the refugee and immigration-related keywords. Results were organized into seven key areas: (1) top keywords, (2) keyword-level sentiment distribution, (3) yearly sentiment trends, (4) sentiment shifts during major immigration events, (5) keyword-level engagement patterns, (6) sentiment differences between immigrant- and refugee-related discourse and (7) geographic distribution of sentiment across the U.S. Together, these analyses offer insights into the emotional tone, reach, and temporal dynamics of migration-related discourse across social media platforms.

Which immigration-related keywords are most prevalent?

### 3.1. Top keywords in migration discourse

To identify the dominant themes in migration-related discourse, we examined the most frequently mentioned keyword groups across all platforms. Table 1 lists the top 20 keywords for each platform, along with sentiment distributions (e.g., negative, neutral, and positive). To improve readability, terms were color-coded consistently across platforms: Blue (general terms), Pink (illegal status), Green (legal status), Purple (deportation and enforcement), and Yellow (protective reforms).

Platform-level differences revealed unique patterns in keyword usage and sentiment. For U.S. based posts on Twitter, “Immigrants” (23.0 %) and “Immigration” (15.3 %) were most frequently mentioned, while “Deportation” (48.4 % negative) and “Border wall” (50.0 % negative) elicited the highest levels of negative sentiment. Conversely, “ICE” (8.0 %), “Sanctuary city” (6.2 % positive), and “Dreamers” (4.3 % positive) reflected relatively higher positivity.

U.S.-based Facebook discussions were dominated by “Immigrants” (20.9 %) and “Migration” (14.5 %). Negative sentiment peaked around “Illegal immigrant” (40.2 %) and “Border patrol” (38.8 %). However, the platform displayed a significant proportion of neutral sentiment across most terms, notably highest for “Temporary Protected Status (TPS)” (81.0 %) and “Undocumented” (80.4 %). While overall positive sentiment remained low, it was comparatively higher for “Dreamers” (14.5 %).

Globally, on Bluesky, “Immigrants” (17.5 %) and “Deportation” (13.7 %) were most prevalent. Negative sentiment was strongest for “Our Country Back” (45.8 %) and “Deportation” (43.1 %), while neutral sentiment predominated, especially for “Sanctuary city” (83.4 %) and “Migration” (82.3 %). Positive sentiment was modest, peaking for “Green Card” (11.4 %) and “Dreamers” (9.3 %).

Across Twitter, Facebook, and Bluesky, “Immigrants” consistently emerged as the most frequent keyword. Other widely discussed terms included “Immigration,” “Illegal,” “Sanctuary city,” “Migrants,” “Refugees,” “Illegal immigrant,” “Undocumented,” “Deportation,” “Asylum,” “Dreamers,” and “Citizenship/Naturalization,” although their rankings varied. Policy and legal status keywords, such as “Sanctuary city” and “Dreamers,” generally showed more positive or neutral sentiment, with “Dreamers” standing out most prominently on Facebook (14.5 %). Neutral sentiment dominated immigration-related discourse online, pointing to a greater prevalence of informational or descriptive content. Additionally, variation in keyword rankings and sentiment distributions highlighted how platform-specific communities have shaped distinct narratives around immigration.

Does sentiment change over time, and do we see spikes in sentiment after immigrant-related events?

### 3.2. Sentiment distribution by keyword group

**Fig. 1.**
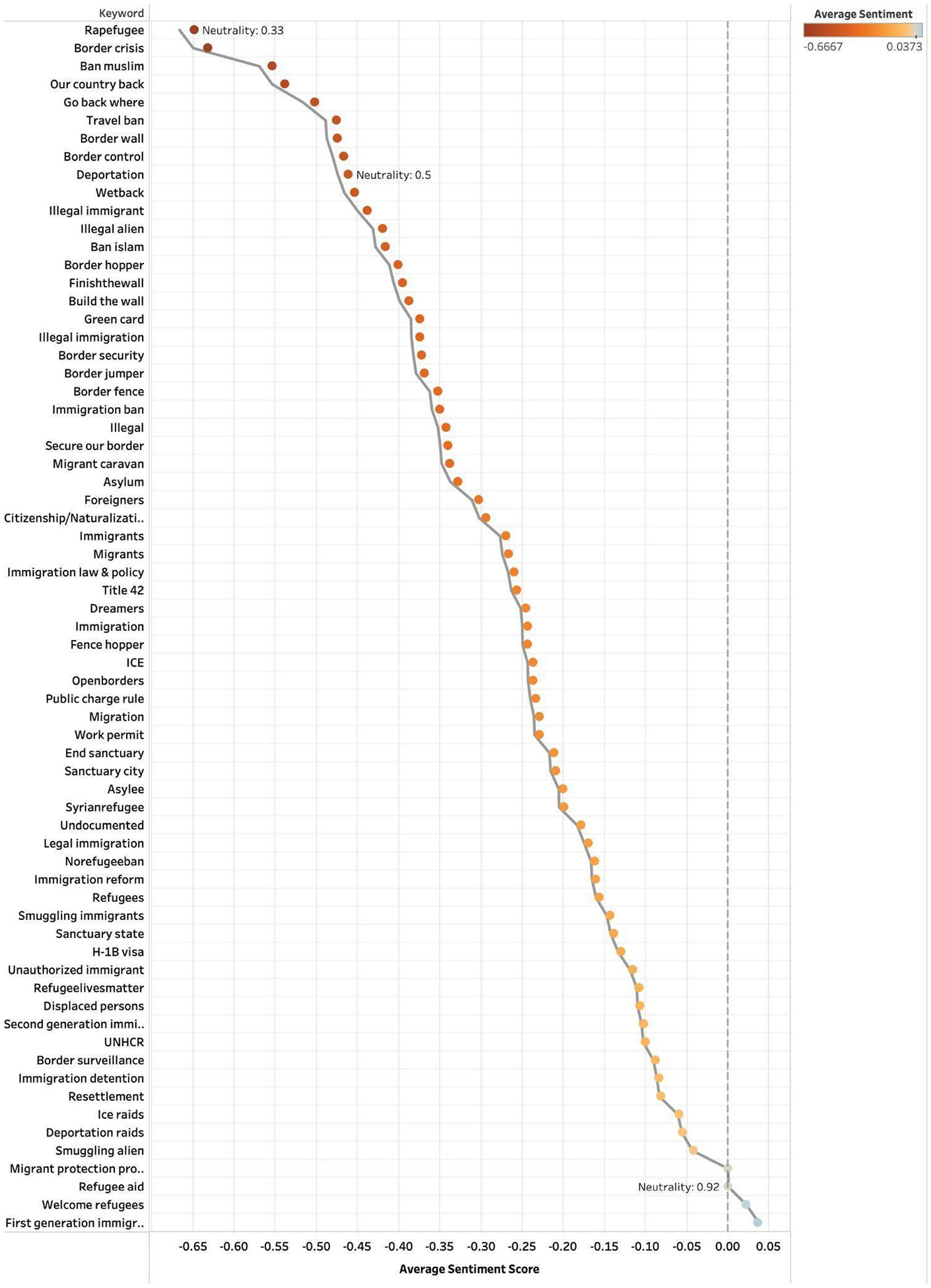
Twitter sentiment scores by grouped keywords (2014–2023).

**Fig. 2.**
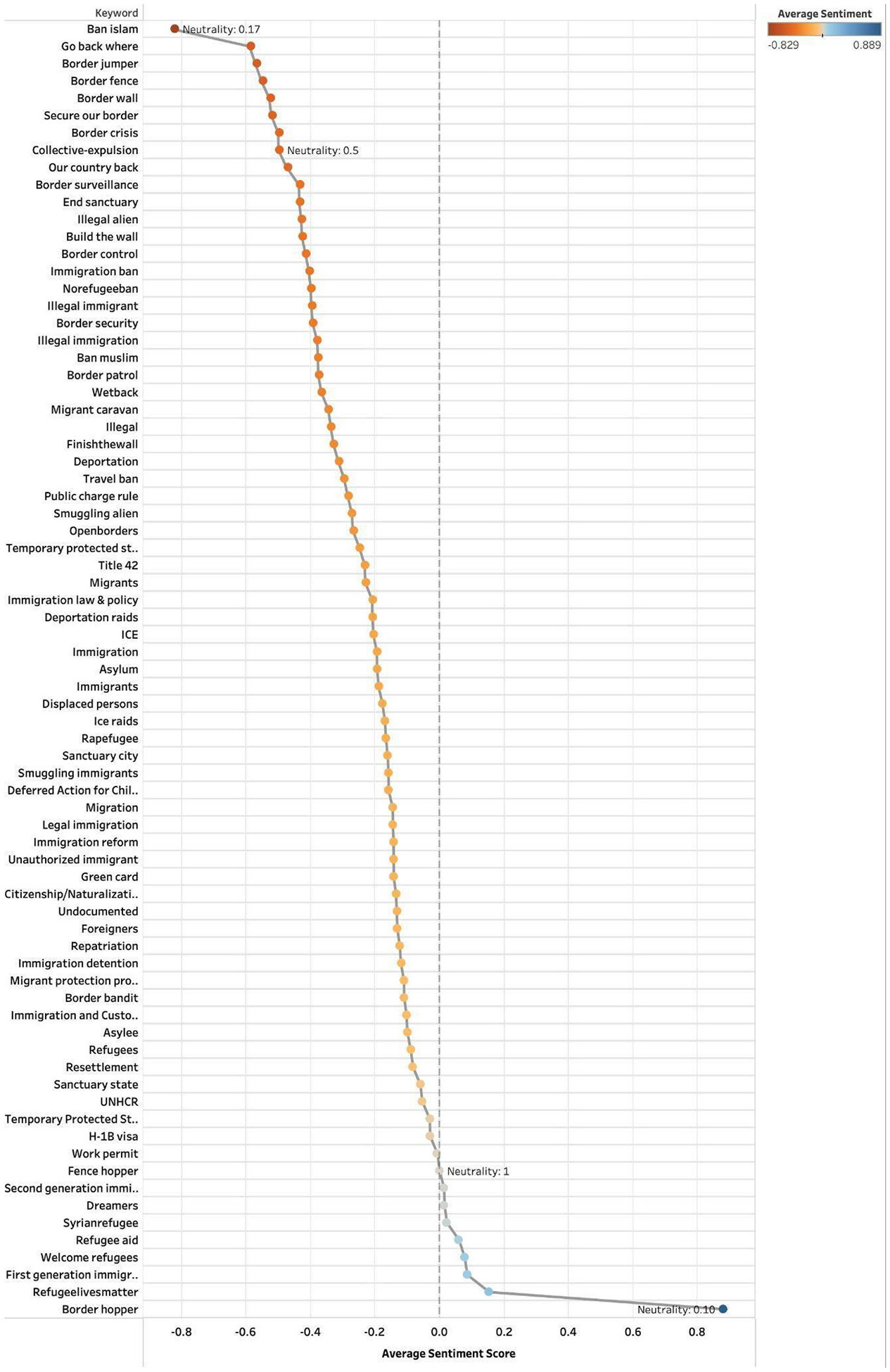
Facebook sentiment scores by grouped keywords (2014–2024).

**Fig. 3.**
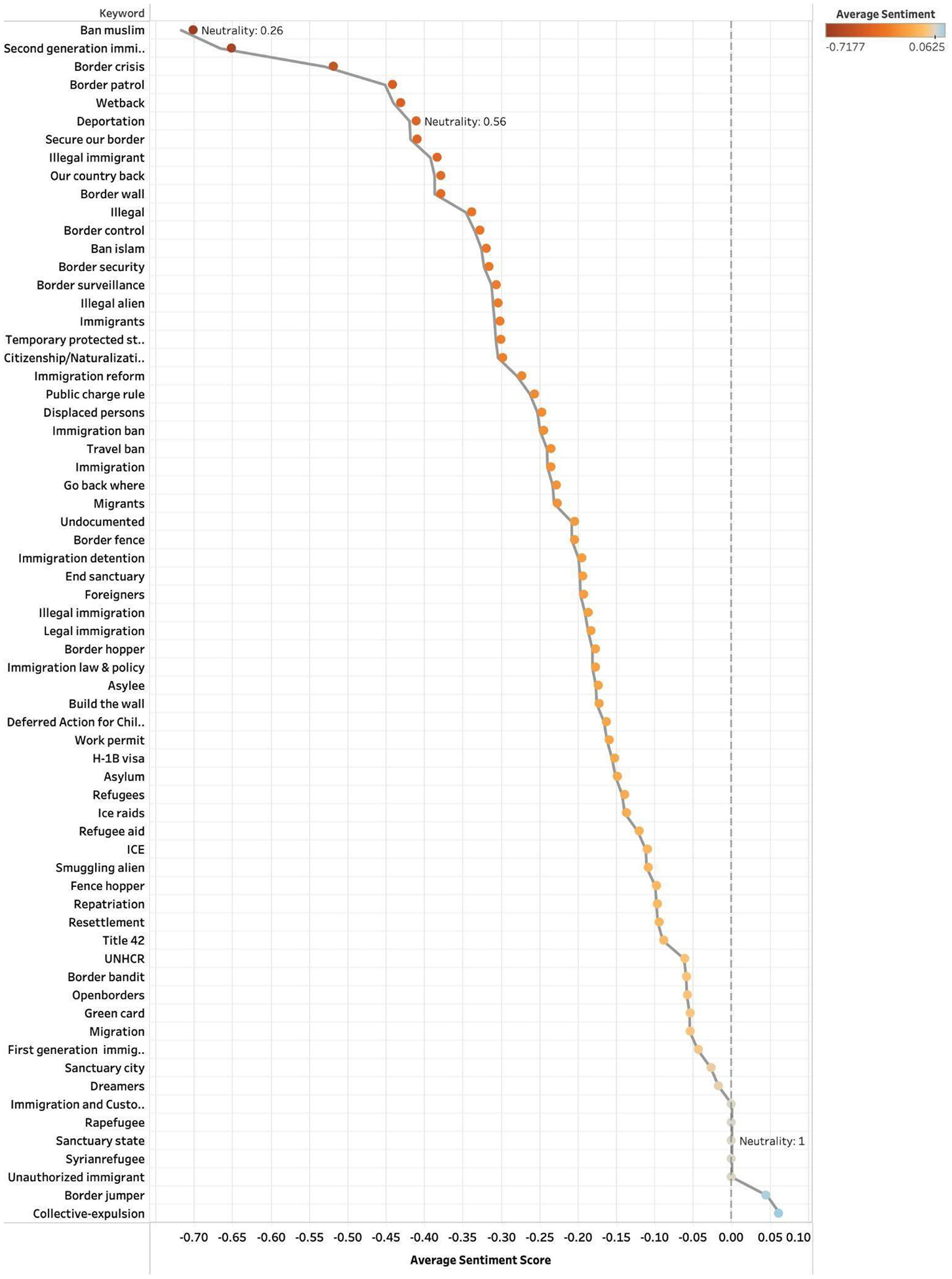
Bluesky sentiment scores by grouped keywords (2023–2024).

To assess how immigration-related topics were emotionally framed across platforms, we examined average sentiment scores and neutrality ratios for each keyword group. Figs. 1–3 show results for Twitter (2014–2023), Facebook (2014–2024), and Bluesky (2023–2024). Each point represents a keyword group plotted by its mean sentiment score, allowing us to distinguish between highly polarized and neutral discourse and to identify terms consistently associated with positive or negative sentiment.

On Twitter, emotionally charged and anti-immigrant terms, such as “Rapefugee” (−0.66), “Border crisis” (−0.64), “Ban Muslim” (−0.57), and “Our country back” (−0.55), received some of the most negative sentiment scores, all with low neutrality (e.g., “Rapefugee” at 0.33). In contrast, terms like “Migrant protection program” (0.00) and “Refugee aid” (0.00) were neutral in sentiment, with “Refugee aid” showing a high neutrality ratio of 0.92. Notably, “First generation immigrant” (0.04) and “Welcome refugees” (0.02) were among the few positively scored terms on the platform (Fig. 1).

On Facebook, “Ban Islam” (−0.83) showed the strongest negativity overall, followed by “Go back where” (−0.59), both with low neutrality (e.g., “Ban Islam” at 0.17). In contrast, several terms reflected mild positivity and high neutrality, including “Second generation immigrant” (0.01), “Dreamers” (0.01), “Syrianrefugee” (0.02), “Refugee aid” (0.06), “Welcome refugees” (0.08), “First generation immigrant” (0.09), and “Refugeelivesmatter” (0.15) (Fig. 2).

On Bluesky, sentiment patterns were generally consistent with other platforms. Strong negative sentiment centered on “Ban Muslim” (−0.72), “Second generation immigrant” (−0.66), and “Border crisis” (−0.53). In contrast, humanitarian and policy-related terms like “Dreamers” (−0.01), “Syrian refugee” (0.00), and “Sanctuary state” (0.00) were among the most neutrally scored keywords(Fig. 3).

Across all three platforms, enforcement-related and exclusionary rhetoric terms, such as “Ban Muslim,” “Border crisis,” and “our country back” consistently appeared among the most negatively scored keywords, with low neutrality indicating emotionally charged discourse. “Rapefugee” and “Ban Muslim” were most negative on Twitter and Bluesky respectively, while “Ban Islam” stood out on Facebook with both the highest negative sentiment (−0.83) and lowest neutrality (0.17).

In contrast, humanitarian and policy-related terms such as “Refugee aid,” “Syrianrefugee,” and “Welcome refugees” had high neutrality ratios (above 0.70). While the general sentiment structure for enforcement-related posts was negative and neutral-to-positive for humanitarian terms, intensity of sentiment and specific term rankings differed across platforms, highlighting unique discourse cultures.

### 3.3. Yearly trends in sentiment over time

**Fig. 4.**
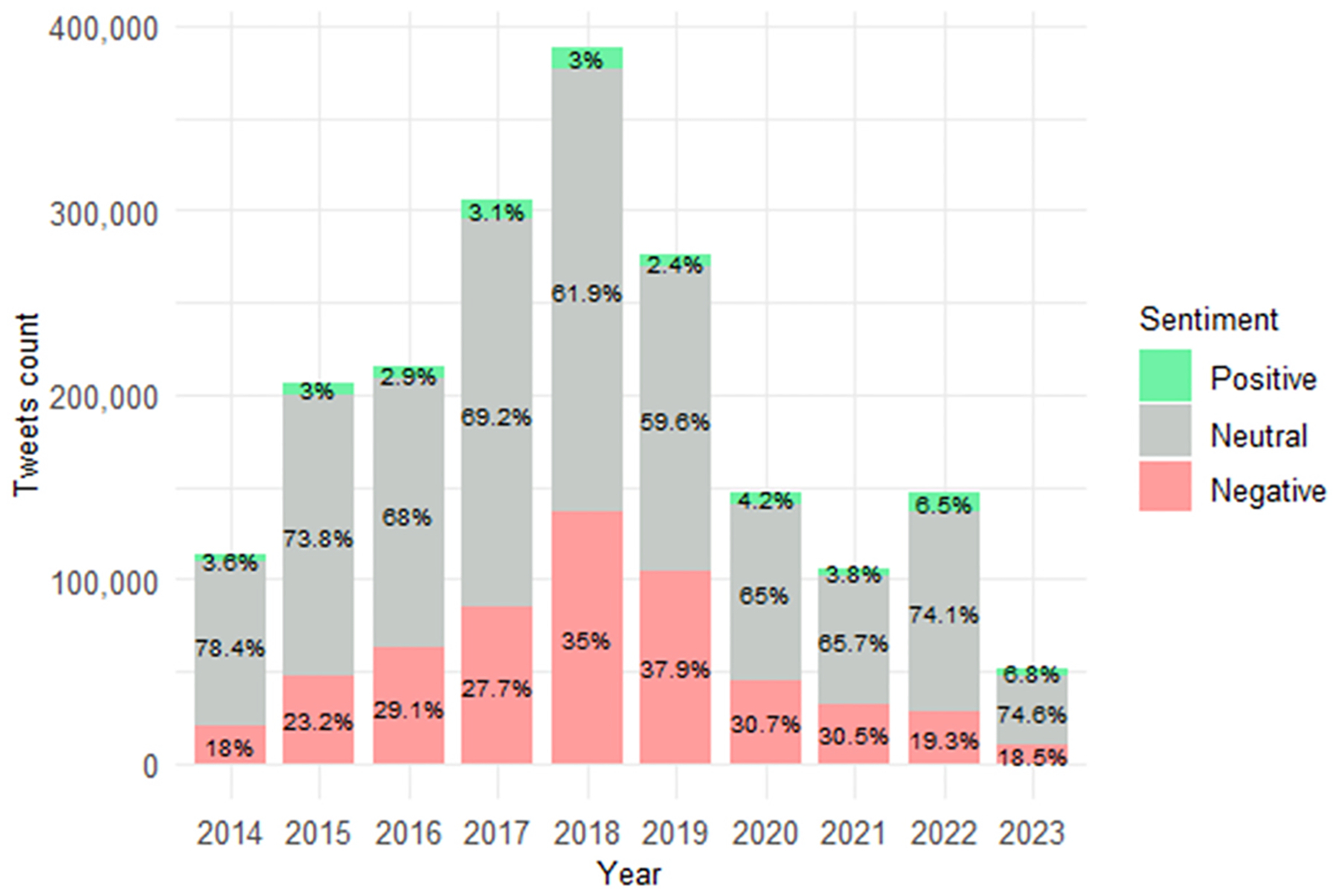
Yearly distribution of sentiment of refugee and immigrant keywords on Twitter (2014–2023).

**Fig. 5.**
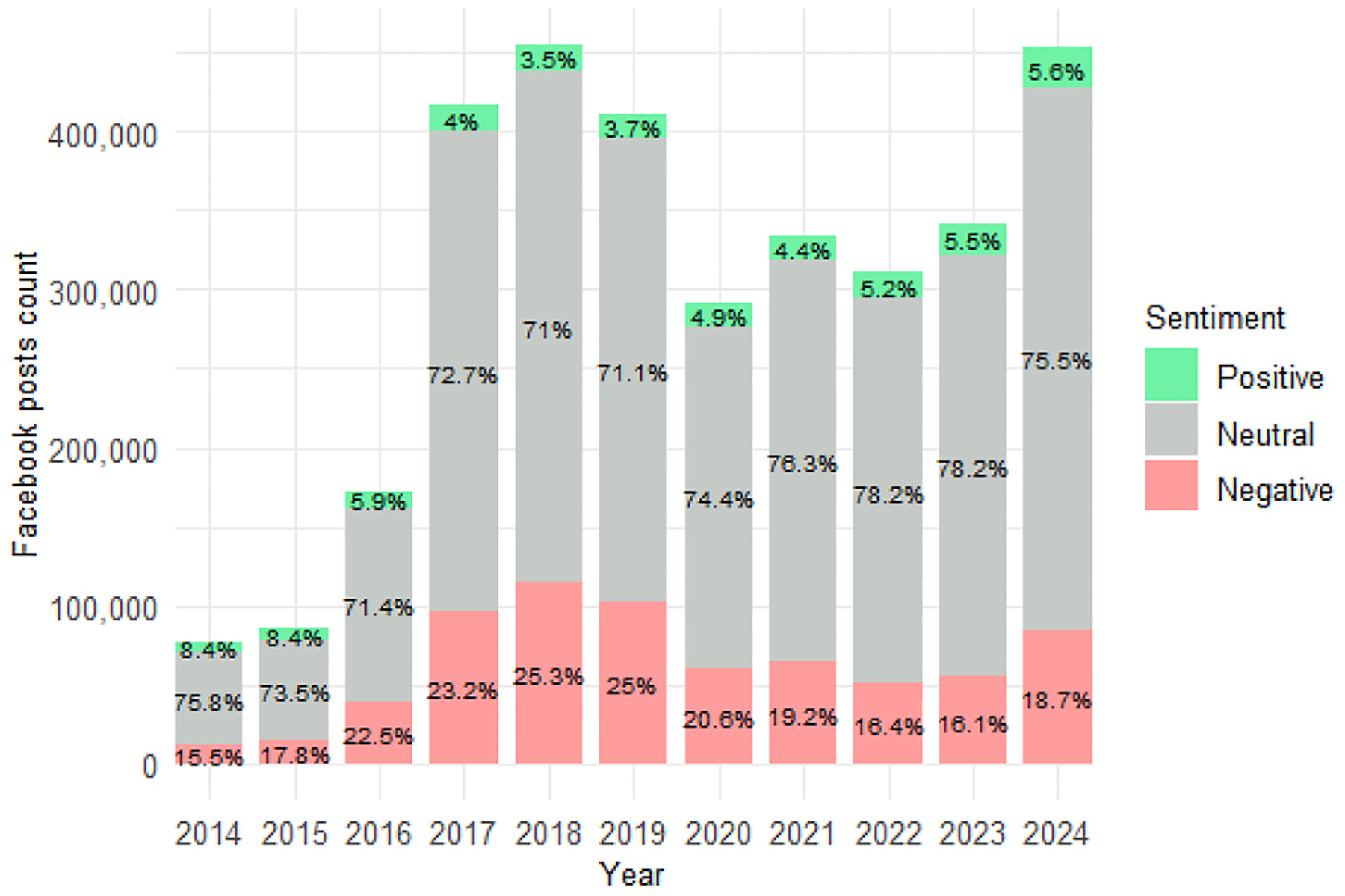
Yearly distribution of sentiment of refugee and immigrant keywords on Facebook (2014–2024).

**Fig. 6.**
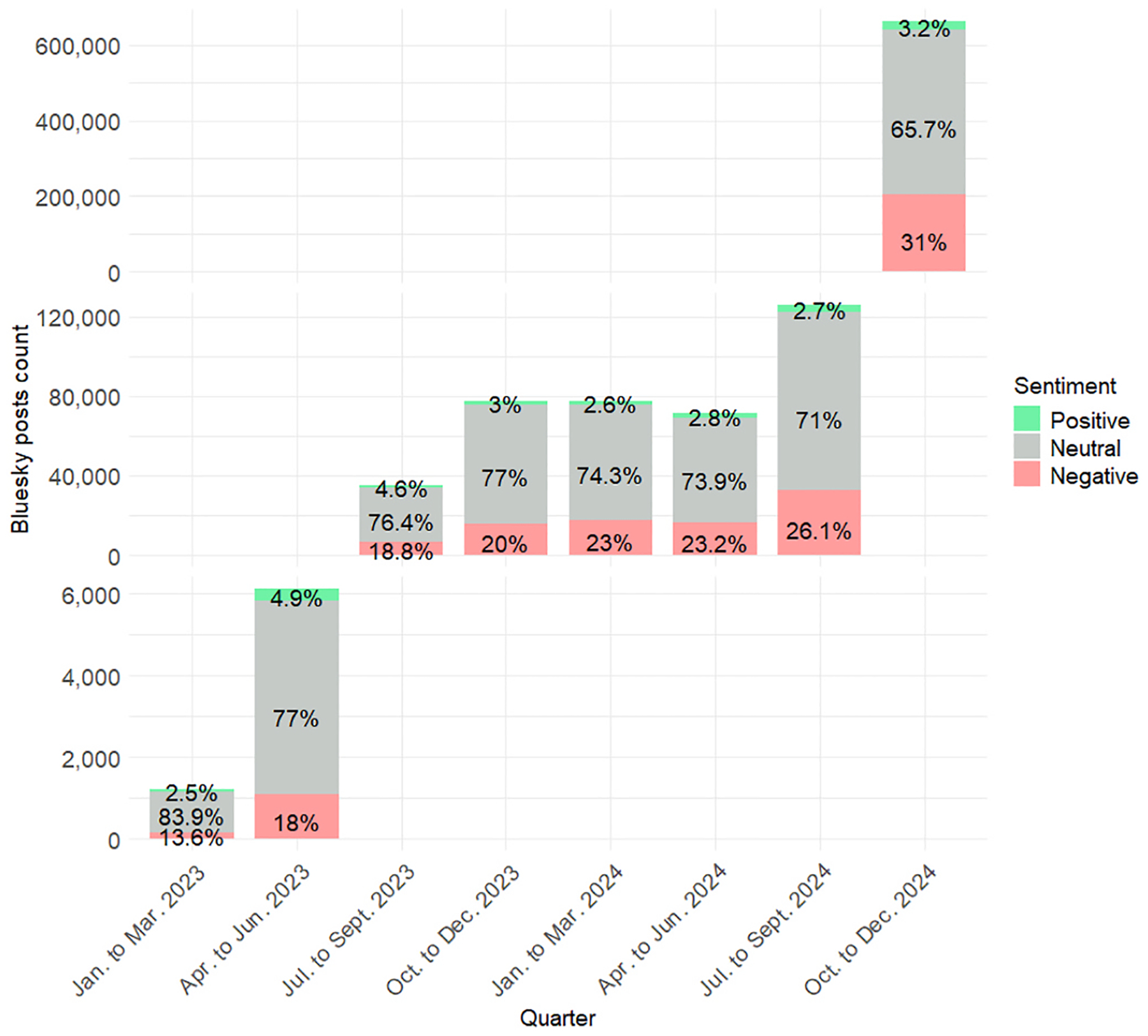
Yearly distribution of sentiment of refugee and immigrant keywords on Bluesky (2023–2024)* *Bluesky experienced rapid increases in usage over time. To display the distribution of sentiment, different axes were used for January–June 2023, July 2023–September 2024, and October–December 2024.

To examine how sentiment of posts referencing refugees and immigrants has evolved over time, we analyzed yearly trends in positive, negative, and neutral sentiment for each platform. Figs. 4–6 display the distribution of sentiment categories across Twitter (2014–2023) and Facebook (2014–2024), while Bluesky trends cover 2023–2024 due to its recent launch.

Twitter, Facebook, and Bluesky each reveal distinct yet overlapping patterns in immigration-related discourse over time. On Twitter, sentiment shifted markedly over 2014–2023. Negative sentiment rose steadily between 2014 and 2019, peaking at 37.9 % in 2019, while neutral sentiment declined from 78.4 % to 59.6 %. Positive sentiment remained low, never exceeding 3.6 %. Tweet volume also increased during this time, suggesting heightened engagement in migration-related discussions. From 2020 onward, sentiment patterns reversed: negative sentiment declined to 18.5 % by 2023, neutral sentiment rebounded, and positive sentiment more than doubled, reaching 6.8 % (Fig. 4).

Facebook followed a similar trajectory, except for 2019, where negative sentiment dropped slightly as positive and neutral sentiment saw small increases. Between 2016 and 2019, negative sentiment remained elevated at 22–25 %, while neutrality hovered around 71–73 %. After 2019, negative sentiment declined to 16.1 % by 2023, before a slight uptick in 2024. Positive sentiment rose to 5.6 %, and neutral sentiment peaked at 78.2 %, indicating a shift toward more balanced discourse (Fig. 5).

Bluesky discourse in early 2023 was largely neutral, with 83.9 % of posts classified as such. Over the next year, sentiment polarized: negative sentiment climbed from 13.6 % in January–March 2023 to 31 % by October–December 2024, while neutral sentiment declined to 65.7 % and positive sentiment remained low (Fig. 6). Despite platform-specific variations, both Facebook and Twitter reflect a broader pattern: high negativity and reduced neutrality in the late 2010s, followed by more neutral and positive discourse in the early 2020s. These findings underscore how both broader social context and platform-level structures shape public sentiment around immigration.

### 3.4. Sentiment shifts during major immigration events

**Fig. 7.**
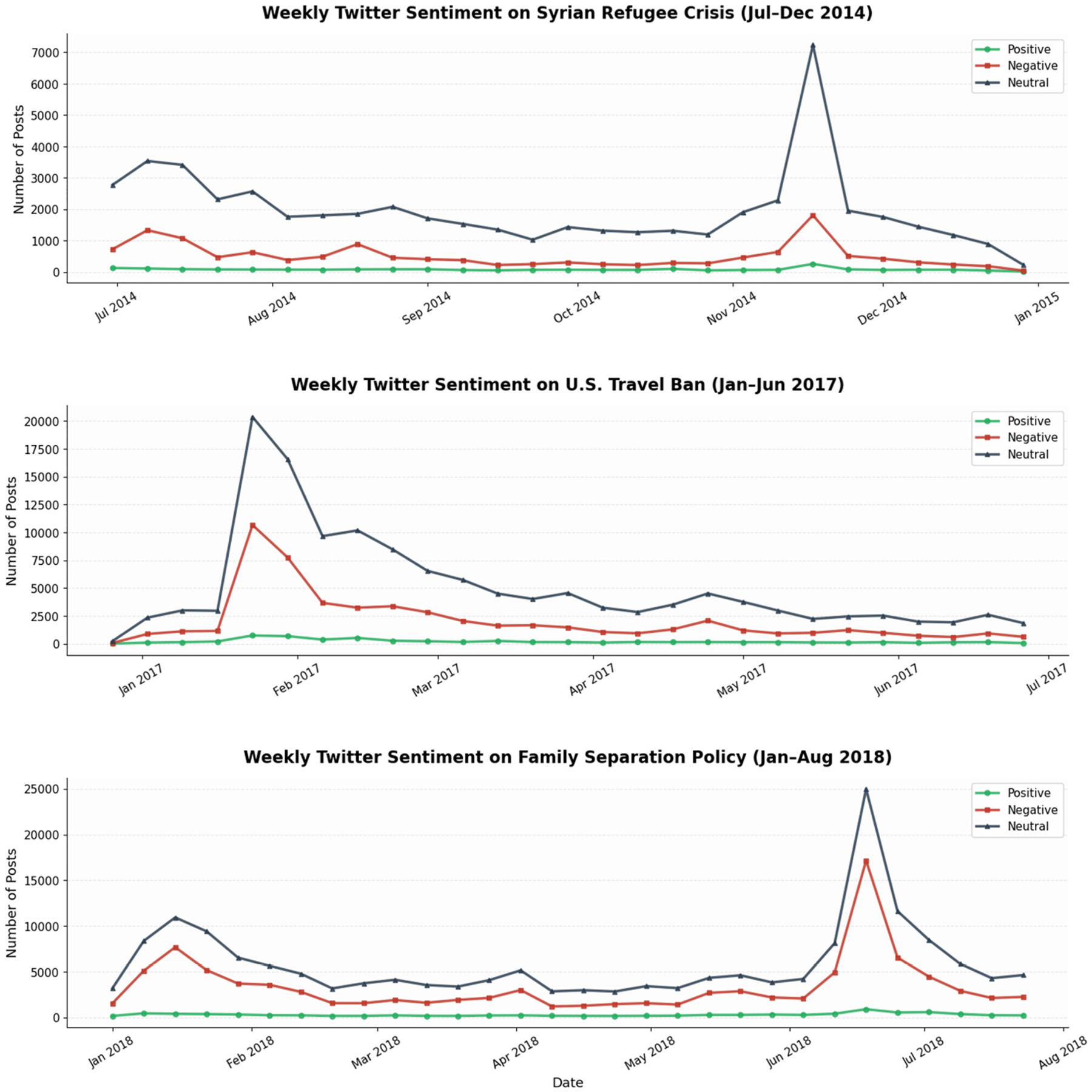
Temporal sentiment trends on Twitter.

**Fig. 8.**
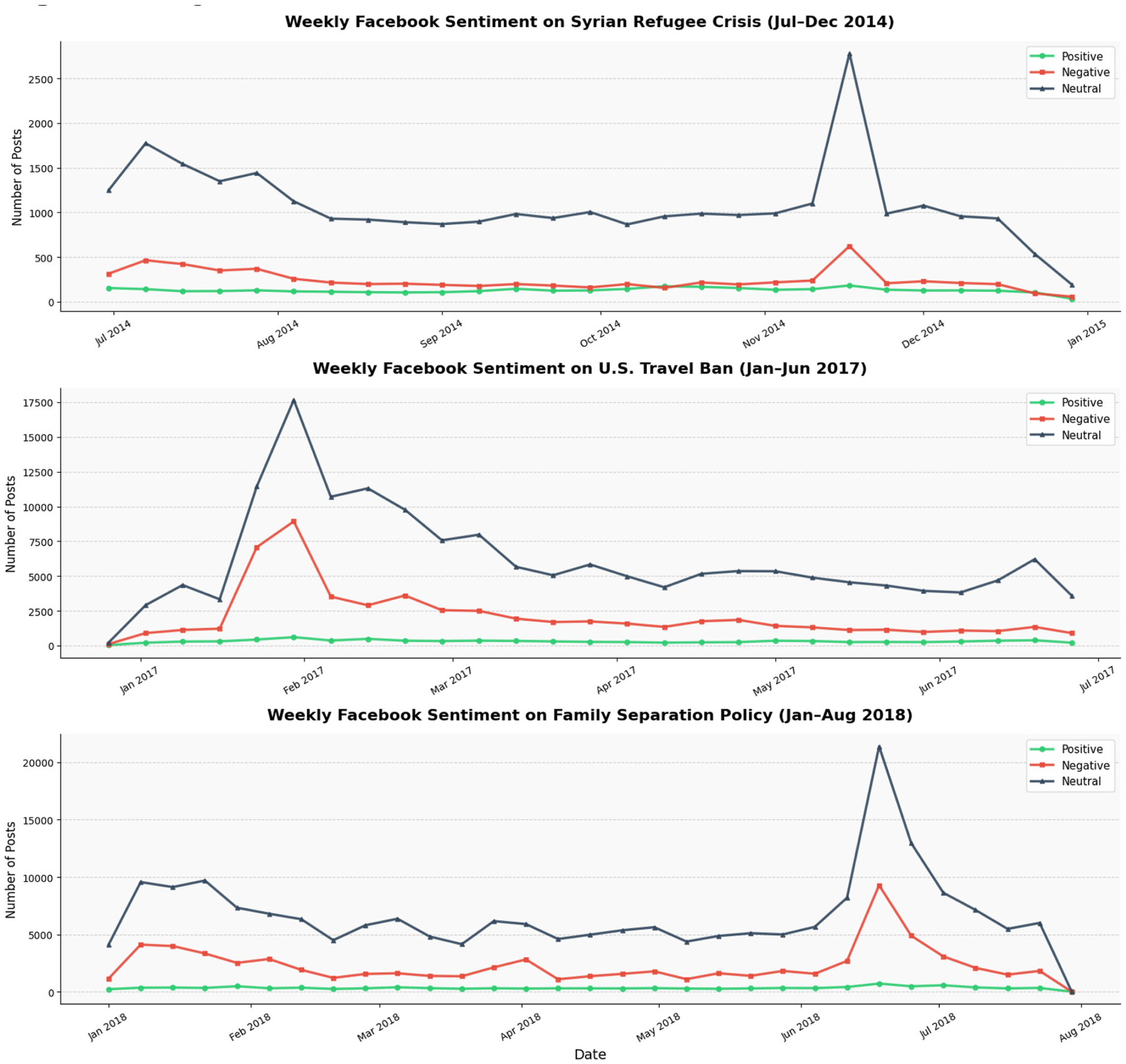
Temporal sentiment trends on Facebook.

To explore how public sentiment responds to key immigration-related events, we analyzed temporal trends around three major moments: the Syrian refugee crisis (2014), the U.S. travel ban (2017), and the family separation policy (2018). Figs. 7 and 8 illustrate monthly sentiment distributions on Twitter and Facebook during these events.

On Twitter, all three events were associated with spikes in user activity and shifts in sentiment. Neutral sentiment dominated overall, but negative sentiment surged dramatically around policy announcements and media coverage. In late November 2014, posts rose sharply during the escalation of the Syrian refugee crisis, when Syrians became the largest global refugee population (UNICEF, 2020). In late January 2017, the announcement of the U.S. travel ban corresponded with a sharp increase in negative sentiment and overall post volume, suggesting immediate and emotionally charged public reactions. Similarly, the rollout of the family separation policy in mid-2018 brought a substantial rise in negative tweets (Fig. 7). The 2018 Human Rights Watch policy was part of the U.S. “zero tolerance” approach, aimed to deter illegal immigration but provoked widespread debate due to its toll on families and lack of reunification infrastructure (Short, 2022). Across all events, positive sentiment remained minimal.

Facebook patterns broadly mirrored those on Twitter with neutral sentiment dominating and negative sentiment increasing during moments of political controversy. During the Syrian refugee crisis in late 2014, post volume rose in November, and negative sentiment slightly outpaced positive sentiment, though both remained relatively low. The U.S. travel ban in early 2017 saw increases in both neutral and negative posts, peaking in February. Similar to Twitter, the overall rise in negativity was more gradual. The family separation policy in mid-2018 led to a substantial rise in negative sentiment, peaking in late June, alongside national media coverage. Across all events, positive sentiment remained low, similar to Twitter (Fig. 8).

Across both Twitter and Facebook, immigration-related events were associated with increased engagement and heightened negative sentiment, particularly during major policy announcements and media focus. The timing of sentiment spikes was closely aligned across platforms; for instance, both saw peak activity and negativity around the Syrian refugee crisis (late 2014), the U.S. travel ban (early 2017), and the family separation policy (June 2018). Together, these findings reveal how immigration-related discourse on social media closely mirrors real-world policy developments and public controversies.

Which keywords spark more engagement, and how do these patterns vary across platforms?

### 3.5. Keyword performance and user engagement

**Fig. 9.**
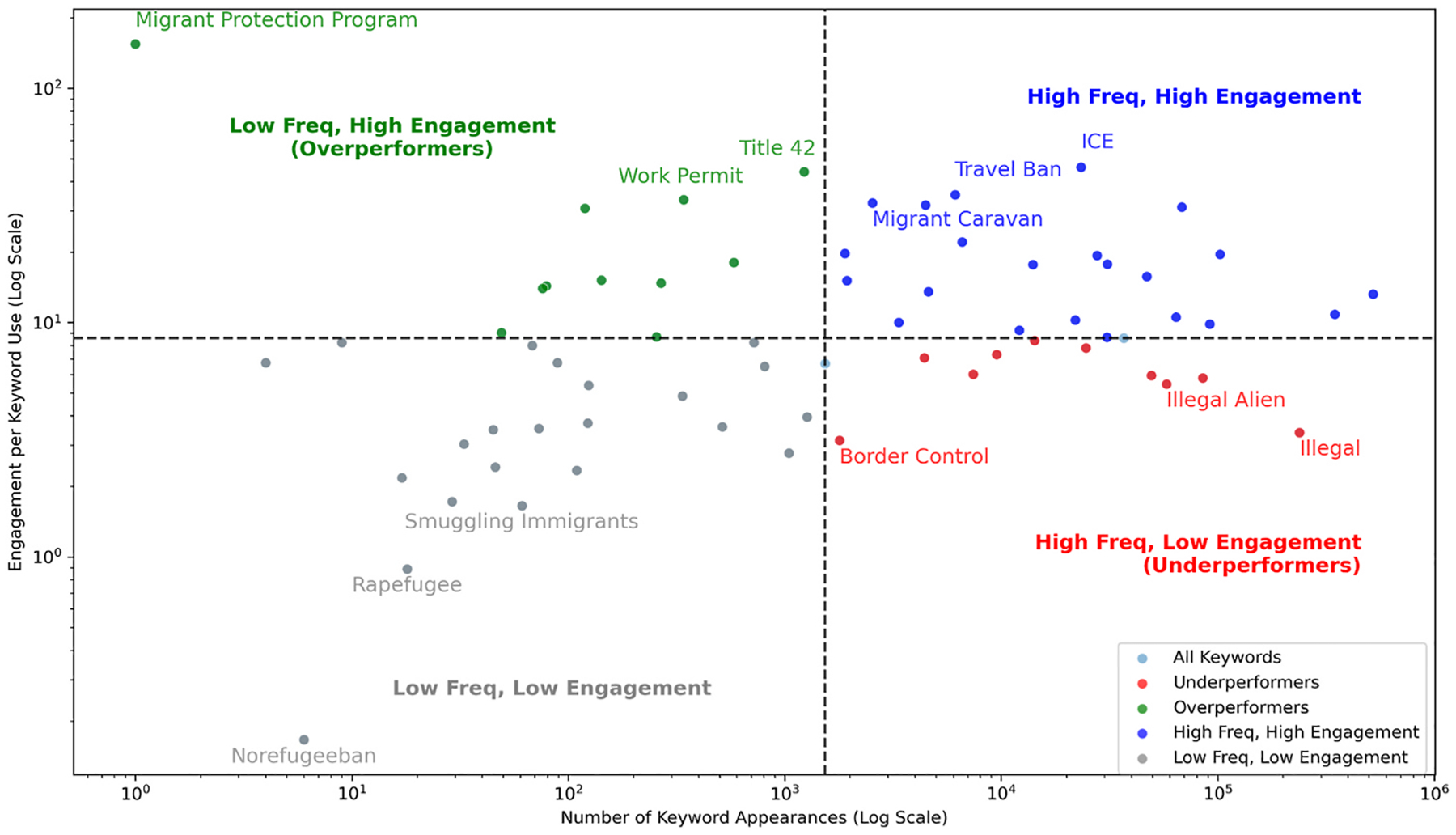
Keyword performance vs. engagement on immigration/refugee discourse (2014–2023) on Twitter.

**Fig. 10.**
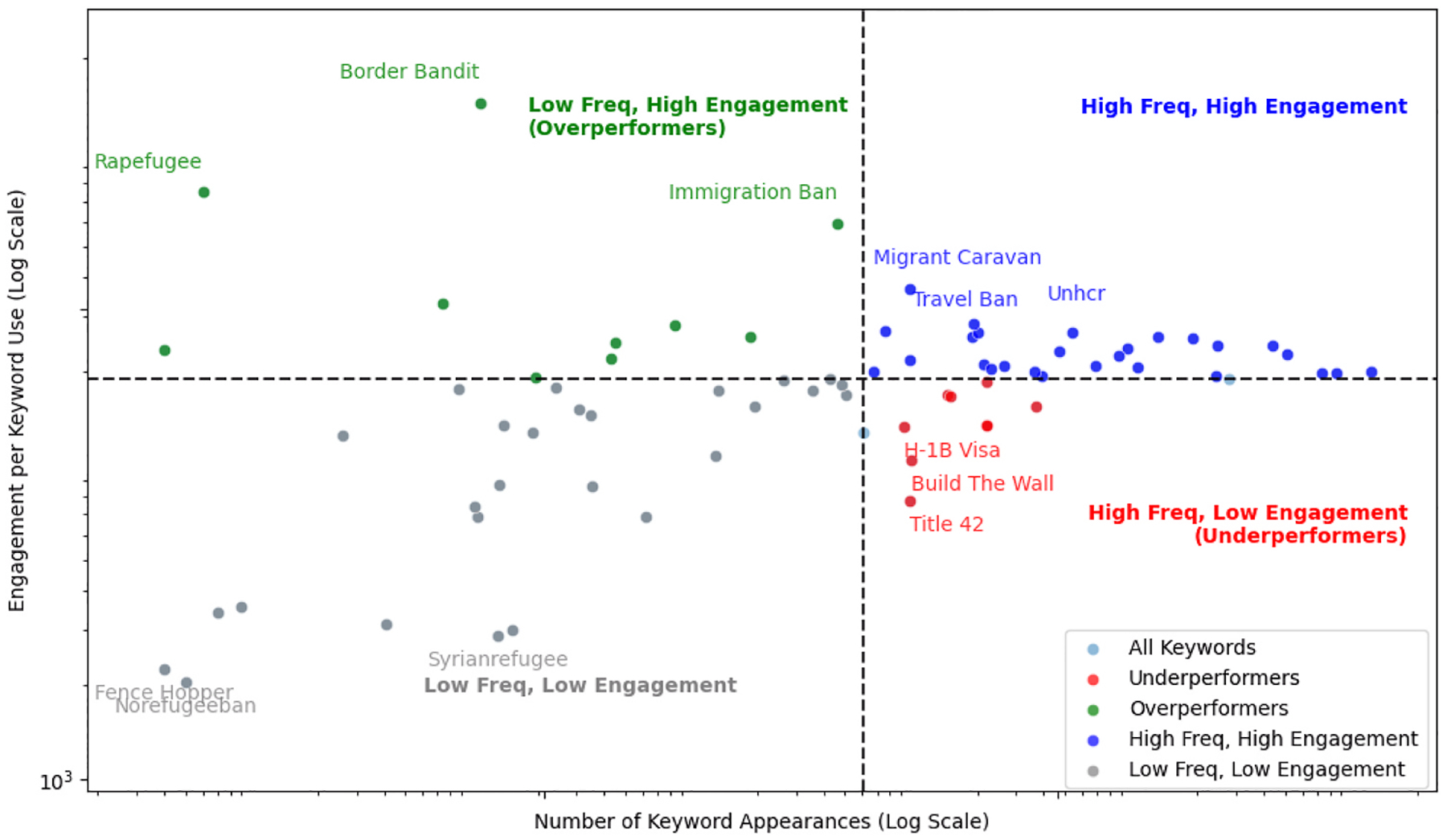
Keyword performance vs. engagement on immigration/refugee discourse (2014–2024) on Facebook.

**Fig. 11.**
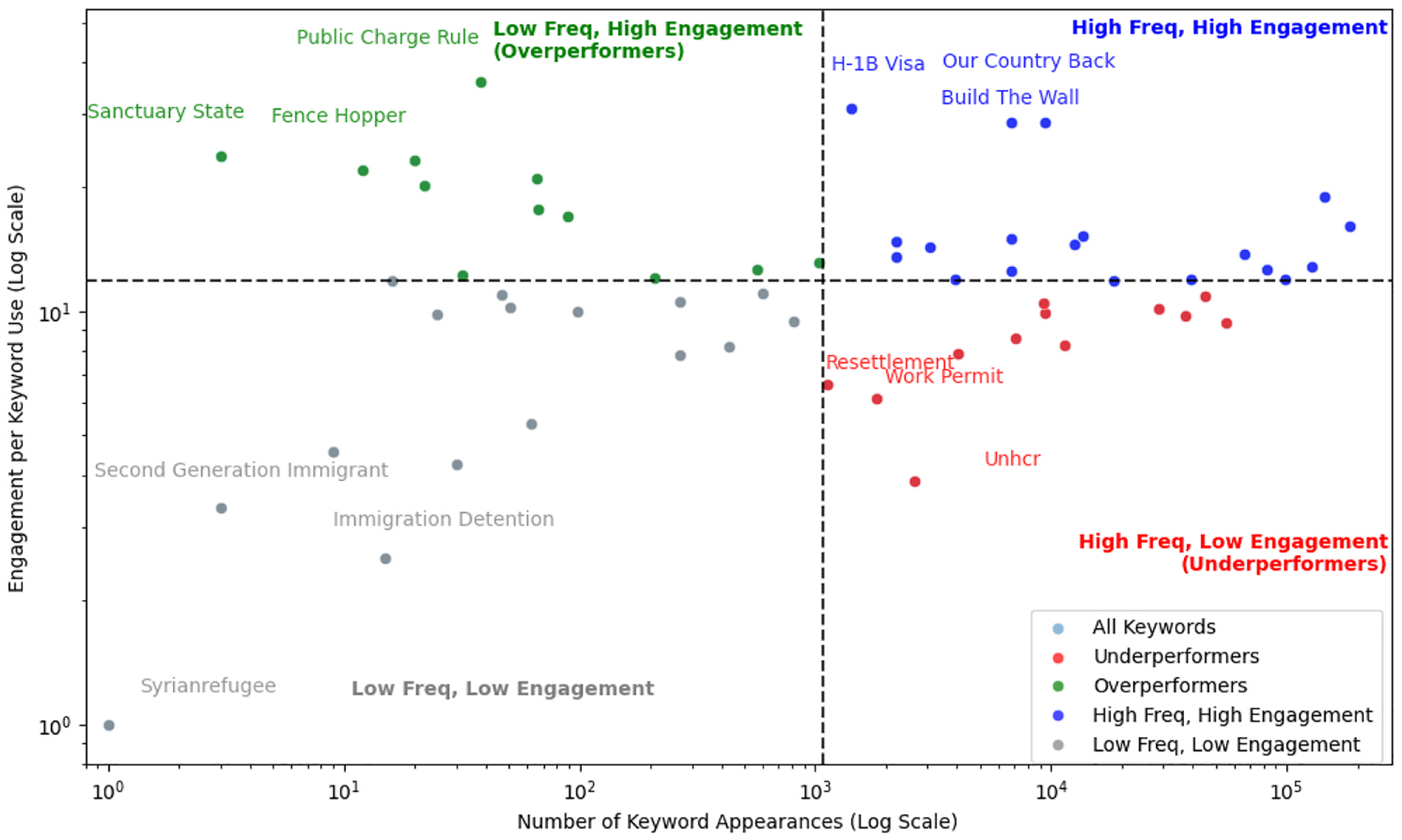
Keyword performance vs. engagement on immigration/refugee discourse (2023–2024) on Bluesky.

To understand how immigration-related keywords captured public attention, we analyzed user engagement across Twitter (2014–2023), Facebook (2014–2024), and Bluesky (2023–2024). Engagement was defined by platform-specific user engagement metrics, aggregated at the keyword group level. By comparing engagement per keyword frequency with keyword frequency, we identified which terms generated disproportionate interaction. Figs. 9–11 depict these relationships. Each keyword is plotted on a log-log scale, with axes representing frequency and engagement per frequency, and divided into four quadrants based on median values.

On Twitter, several keywords emerged as overperformers, generating high engagement despite low frequency including “Migrant Protection Program,” “Work Permit,” and “Title 42.” In contrast, frequently used terms such as “Border Control” and “Illegal Alien” were categorized as underperformers, while appearing frequently but receiving lower interaction per mention. Niche keywords like “Smuggling Immigrants” and “Rapefugee” exhibited both low frequency and engagement. Meanwhile, “Migrant Caravan,” “Travel Ban,” and “ICE” occupied the high frequency, high engagement quadrant, suggesting broad interest and consistent interaction (Fig. 9).

On Facebook, overall patterns resembled Twitter, with “Migrant Caravan” and “Travel Ban” consistently appearing in the high frequency, high engagement quadrant, reaffirming their strong cross-platform resonance. However, platform-specific differences surfaced. For instance, “Title 42” an overperformer on Twitter, underperformed on Facebook despite its visibility. By contrast, “Rapefugee” which received low engagement on Twitter, emerged as an overperformer on Facebook, eliciting high engagement despite its low usage, underscoring how polarizing terms resonate differently by platform. Other over performers included “Border Bandit” and “Immigration Ban.” By contrast, “Norefugeeban” consistently occupied the low frequency, low engagement quadrant, thus highlighting its limited reach (Fig. 10).

On Bluesky for global immigrant-related posts, terms such as “Public Charge Rule,” “Fence Hopper,” and “Sanctuary State” emerged as overperformers. By contrast, frequently appearing terms like “Resettlement,” “Work Permit,” and “UNHCR” generated relatively low engagement while having high frequency. Keywords such as “H-1B,” “Our Country Back,” and “Build the Wall” were widely discussed and highly engaging. Finally, niche terms including “Second Generation Immigrant,” “Immigrant Detention,” and “Syrian Refugee” were infrequent and drew minimal interaction (Fig. 11).

Patterns of keyword engagement diverged sharply from Twitter, with no overlap in high or low performing terms. While “Build the Wall” appeared in the high frequency, high engagement quadrant on Bluesky, indicating strong user responsiveness, it underperformed on Facebook, where it ranked high in frequency but low in engagement. Conversely, “UNHCR” performed well on Facebook (high frequency and engagement) but showed markedly lower engagement on Bluesky despite similar usage levels. A point of consistency emerged with “Syrian Refugee,” which occupied the low frequency, low engagement quadrant on both Facebook and Bluesky, signaling limited resonance regardless of platform. Overall, Bluesky’s keyword landscape follows a distinct trajectory from established platforms, shaped by its newer user base and unique patterns of discourse.

Do refugee-related discussions display similar or distinct sentiment patterns compared to immigrant-related discussions? How is sentiment for posts referencing refugees and immigrants distributed geographically across the United States?

### 3.6. Sentiment differences between immigrant and refugee discourse

**Fig. 12.**
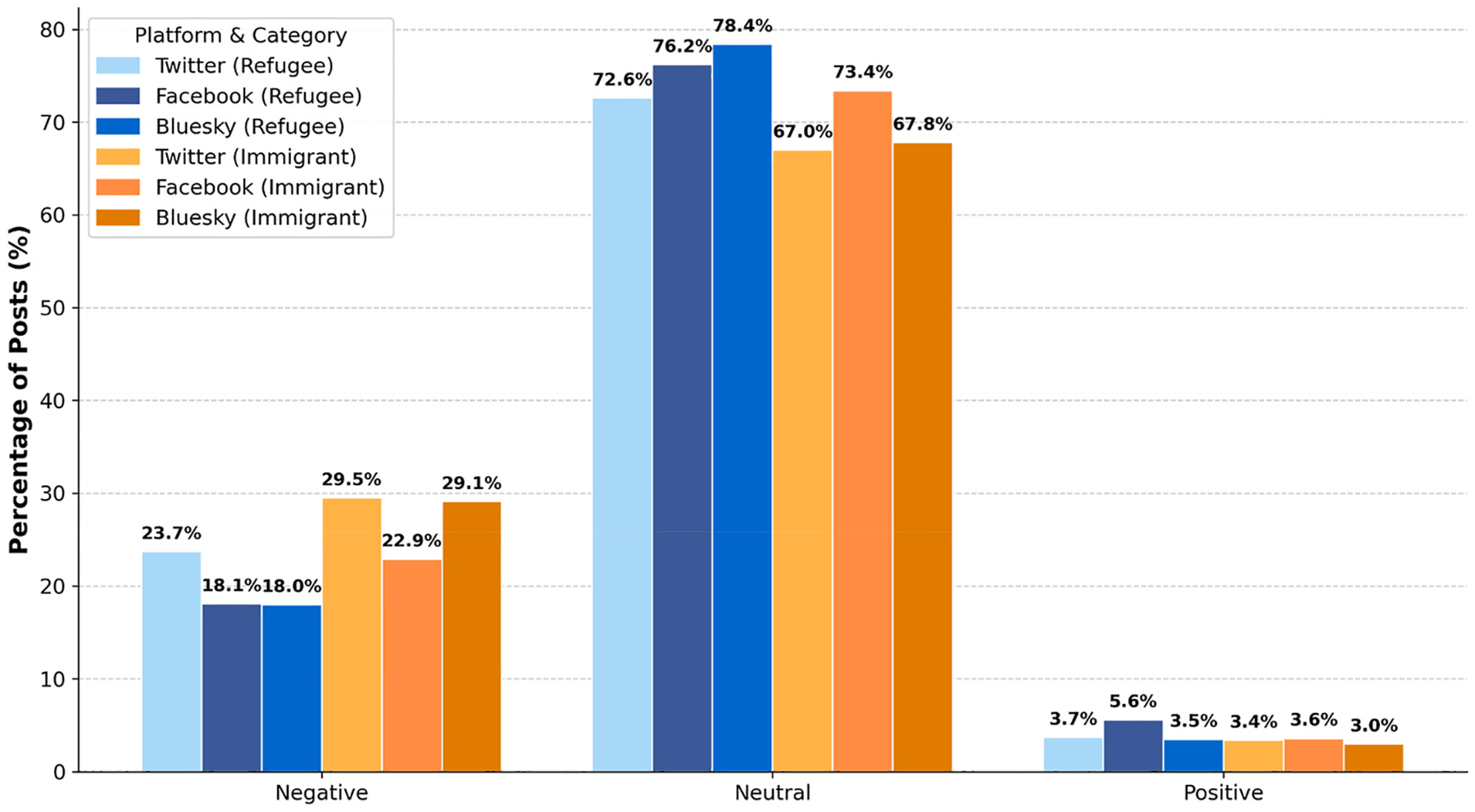
Sentiment distribution of refugee and immigrant-related posts by social media platform.

To further explore variation in sentiment, discourse specifically referencing refugees were examined compared to posts referencing immigrants broadly. Across all three platforms, neutral sentiment dominated, but posts referencing immigrants consistently carried more negativity than refugee-related posts. Although Facebook generates the largest volume of posts, its sentiment profile aligns with Bluesky and Twitter: content referencing refugees remains comparatively neutral with pockets of positive engagement, while content referencing immigrants elicits a noticeably higher proportion of negative posts (Fig. 12).

### 3.7. Geographic distribution of sentiment comparing refugee and immigrant related discussions

To explore regional patterns in public sentiment, we mapped average sentiment scores using Twitter data from 2014 to 2023 across U.S. states. Three maps were generated: one aggregating all keywords, and two disaggregated maps for “immigrant” and “refugee” related keywords, respectively. eFig. 1 and eTable 2 presents the average sentiment scores of posts referencing both groups combined. Sentiment scores range from+1 (positive) to −1 (negative), with 0 as neutral. Overall, sentiment remained predominantly negative nationwide with no state exhibiting a neutral or positive average score during the observed period.

**Fig. 13.**
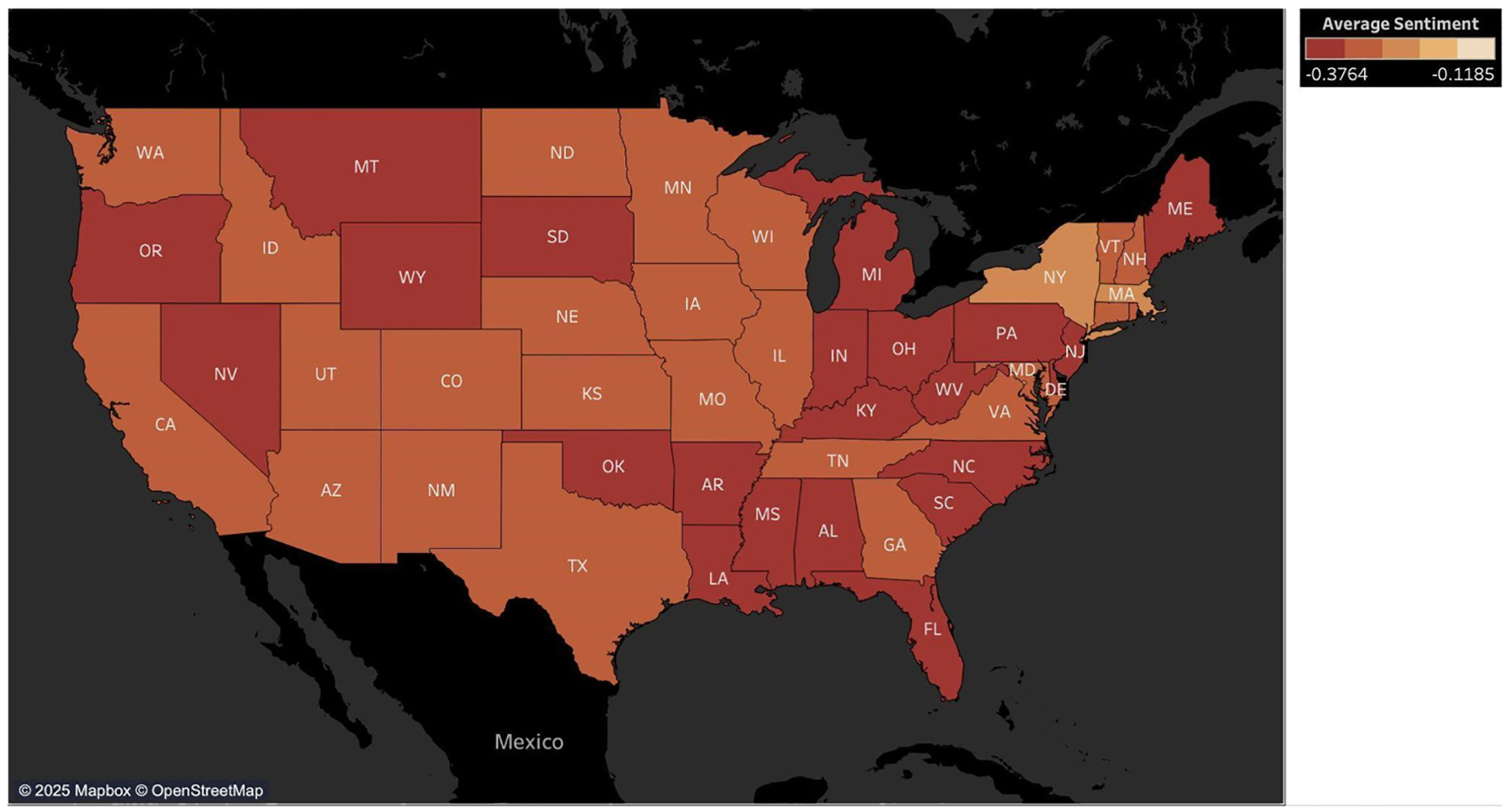
State-level sentiment for immigrant keywords by state (2014–2023) on Twitter.

**Fig. 14.**
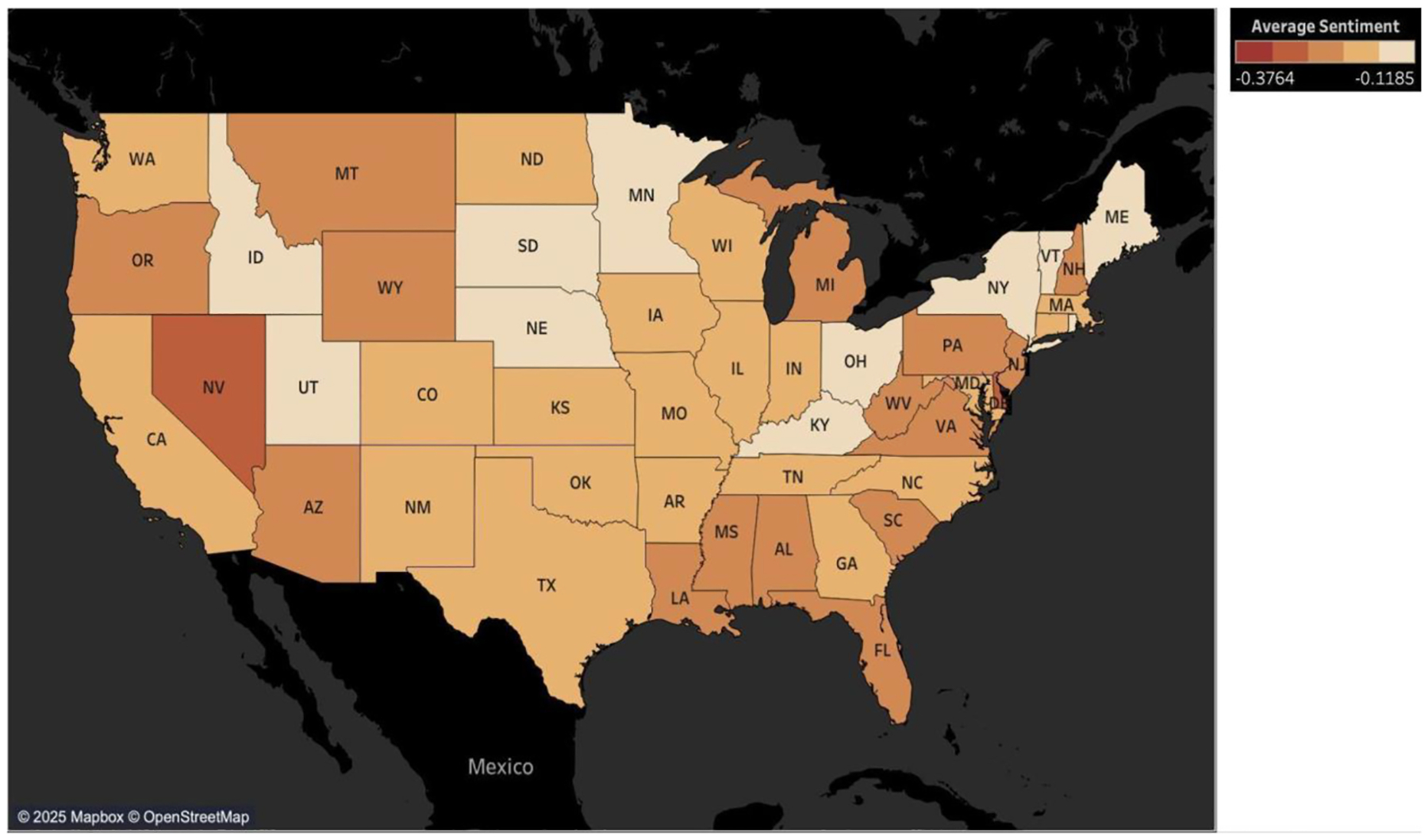
Average sentiment for refugee keywords by state (2014–2023) on Twitter.

A comparison of state-level sentiment (2014–2023) of posts referencing immigrants and refugees revealed differences in tone and intensity of public discourse across the United States. Although sentiment was predominantly negative across all states, posts referencing refugees were consistently less negative than about immigrants, both in magnitude and spatial distribution. The immigrant sentiment map (Fig. 13) is dominated by darker red and deep orange shades, particularly across the South, Northeast, Midwest and parts of the West, indicating widespread and intense negativity. In contrast, the refugee sentiment map (Fig. 14) shows lighter tones across many states, suggesting a more moderate or neutral discourse for posts using refugees-related keywords. For example, in Alabama the average sentiment score for posts using immigrant-related keywords was −0.343 compared to −0.255 for refugee-related keywords. Similarly, North Carolina recorded −0.326 versus −0.182, Illinois showed −0.287 versus −0.171 Even in states with the most negative sentiment for posts referencing immigrants, such as West Virginia, Kentucky, and Oklahoma, sentiment of posts referencing refugees was still markedly less negative. Hawaii, Alaska, and Puerto Rico were excluded from the maps due to their geographic separation from the contiguous U.S., but their sentiment scores follow the same trend. In Hawaii, sentiment for posts referencing immigrants was −0.320, versus −0.202 referencing refugees; in Alaska −0.334 versus −0.286; and in Puerto Rico −0.297 versus −0.188. These cases underscore a nationwide pattern of relatively more favorable public discourse of refugee-related posts. Thus, although immigrants and refugees are frequently grouped together in political and media narratives, public perception distinguishes between them in meaningful ways.

## 4. Discussion

This study used more than 6 million public posts across Twitter (2014–2023), Facebook (2014–2024), and Bluesky (2023–2024) to examine how refugees and immigrants are viewed and discussed online. Our findings reveal patterns in emotion, tone, user engagement, and geolocation distribution surrounding online migrant related conversations. Sentiment of refugees and immigrants-related discourse online is generally neutral, but sharp surges in negative sentiment occurred around major policy events, such as the U.S. travel ban (2017) and the family separation policy (2018). These spikes, while intense, were often brief. Enforcement-related and exclusionary rhetoric terms consistently carried high negative sentiment across platforms, whereas positive sentiment was less common and centered on discussions surrounding “Dreamers” or support for refugees from conflict regions (e.g., “Refugee aid” and “Welcome refugees”). Although platform dynamics varied, Twitter and Facebook exhibited similar temporal trends: heightened negativity in 2010s, gradually shifting toward more balanced or positive conversations in the early 2020s. Bluesky, in contrast, displayed an uptick in negative sentiment following its public release in 2024. These results indicate that online discussions about migration are highly responsive to external events, and differences in immigrant-related discussions vary across social media platforms.

User engagement data demonstrates that keyword popularity does not always correlate with engagement. Frequently used terms (e. g.,“Illegal alien” and “Border Control”) attract moderate engagement, while rare but emotionally charged phrases(e.g., “Rapefugee,” “Our Country Back”) consistently draw disproportionate engagement. Policy-related keywords (“Migrant Caravan,” “Travel Ban,” and “ICE”) also trigger high engagement across social media platforms. Platform differences further shape these patterns. For example, “Build the Wall” appeared frequently and sparked high engagement on Bluesky, it underperformed on Facebook, where it ranked high in frequency but low in engagement. Conversely, “UNHCR” performed well on Facebook (with both high frequency and engagement) but showed markedly lower engagement on Bluesky, specifying platform-specific affordances and community effects.

Geographic analysis further reveals that sentiment and keyword usage are unevenly distributed across regions for immigration-related keywords. Negative sentiment is most intense in parts of the South, West, and Mid-Atlantic Northeast. In contrast, some areas in the New England Northeast and Midwest show relatively less negativity for posts referencing immigrants and refugees. Importantly, across all regions, the public expresses less negative sentiment for posts referencing refugees compared to immigrants. These results reveal the influence of regional context and the importance of distinguishing between immigrant and refugee groups when understanding public online discussions.

Rather than treating digital engagement as uniform, this analysis highlights how platform design and audience composition collectively shape which topics gain traction, how they are framed, and who engages with them. Overall, these insights emphasize the importance of platform-specific approaches when interpreting online public sentiment.

Our findings align with a growing body of literature emphasizing the critical role of framing in shaping public sentiment related to immigration. Across all platforms and U.S. states, refugee-related terms consistently exhibited less negative sentiment than immigrant-related terms. This pattern reinforces the argument advanced by Lee and Nerghes (2018) that the term “refugee” tends to evoke humanitarian and sympathetic associations, whereas “immigrant” is more frequently linked to economic threats or political anxieties. Notably, this framing effect persisted not only during moments of crisis but across the entire decade of analysis, highlighting the enduring influence of discursive labels. Building on earlier work by Grover et al. (2019), who documented short-term sentiment fluctuations around specific U.S. immigration events from 2016 to 2017, including the Muslim Ban and DACA repeal, our study expands the temporal and platform scope. By analyzing migration-related discourse from 2014 to 2024 across Twitter, Facebook, and Bluesky, we provide a longer timescale and cross-platform perspective on how narratives about immigrants and refugees are constructed, sustained, and transformed in the digital public sphere. This broader view also captures the episodic intensification of anti-migration discourse during the COVID-19 pandemic documented by Sievers et al. (2021). We observed pronounced spikes in negative sentiment and posting activity during key flashpoints such as the 2014 Syrian refugee crisis, the 2017 U.S. travel ban, and the 2018 family separation policy. These surges underscore how moments of political and humanitarian significance catalyze heightened activity and emotional polarization online.

Complementing this temporal analysis, our engagement metrics further support the Mendelsohn et al., (2021) argument that emotionally charged, politically framed content garners disproportionate attention. Terms such as “Migrant Caravan” and “Travel Ban” consistently appeared in high-frequency and high-engagement clusters across both Twitter and Facebook. Finally, our cross-platform comparisons deepen insights from Gruzd et al. (2024), whose study focused on anti-refugee discourse on Twitter, demonstrating that discourse dynamics can differ across platforms. For example, while the derogatory term “Rapefugee” attracted limited engagement on Twitter, it experienced far greater amplification on Facebook, indicating how both platform-specific structures and user base mediate the circulation and salience of inflammatory rhetoric.

### 4.1. Strengths and limitations

This study offers several notable strengths that enhance its contribution to the literature on digital immigration discourse. First, the analysis spanning a full decade (2014–2024) enables a long-term perspective on immigration trends, moving beyond the short-term, event-focused analyses that dominate prior work. The inclusion of three distinct social media platforms (i.e., Twitter, Facebook, and Bluesky - a relatively new platform) further advances a comprehensive comparison of platform-specific discourse cultures and temporal trends. The study draws on a large and diverse dataset of over six million publicly available posts, enabling robust analyses and broader relevance of findings. Additionally, the use of a keyword framework comprising 129 terms grouped into 76 thematic categories enables nuanced sentiment and engagement analysis at the topic level. This is complemented by analytical approaches such as the engagement-frequency quadrant framework and geographic sentiment mapping based on Twitter’s location metadata. The integration of a machine learning classifier trained on both public and manually labeled datasets ensures reliable sentiment detection, while the incorporation of a neutrality ratio allows for more accurate interpretation of polarized versus neutral discourse. Lastly, event-based sentiment analysis highlights the utility in capturing public reactions to key immigration policies and moments of heightened media attention, reinforcing the study’s relevance to both social and political contexts.

This study is not without limitations. Our sample represents what people choose to post on Twitter, Facebook, and Bluesky. While these platforms are used by a diverse range of individuals, the views represented do not necessarily mirror those of the general U.S. population. Furthermore, each platform has distinct demographic profiles, features, and policies, and these differences may influence both the composition of users and the study’s findings. These factors should be considered when interpreting our results. While we developed a comprehensive list of refugee and immigrant keywords, social media is dynamic and ever evolving, and this list may omit relevant discussions that use alternate language. Additionally, while our sentiment classification model was capable of identifying general patterns, it may not have completely captured nuanced statements, especially in posts with sarcasm, ambiguous expressions, or emoji symbols. Furthermore, it is possible for a post to convey a negative tone without directing that negativity toward the group being referenced. In many cases, a negative emotional tone did not equate to a prejudiced statement. Our prior research has also shown that prejudiced content can be distinct from overall sentiment (Nguyen et al., 2019). While some negatively-toned posts did reflect racial prejudice or discriminatory beliefs (e.g., “any muslim that raises his hand or voices dislike for something american should be labeled terrorist and deported), others expressed negative sentiment without conveying prejudice (e.g., “there is shortage of lawyers specialization in immigration which fuels problem it hurts everyone even employers”).

Moreover, platforms differ in their data access and data structure. For example, the free Twitter Academic Research API was discontinued in 2023, and Bluesky has emerged recently within the past two years. Although we were able to restrict to the U.S. for Twitter and Facebook, a country-level filter was not available for Bluesky. Regardless of this limitation, Bluesky posts provided valuable insights for immigrant-related discussions on an emerging platform. Potential approaches to identify country-specific posts can occur during the post-processing data stages. For example, machine-learning methods, such as parsing profile location fields, analyzing posting times against time zones, or using named-entity recognition on text may be used to infer a user’s country (Dai et al., 2023). However, these approaches rely on incomplete or noisy metadata, cannot be applied to all users, and are prone to misclassification errors, leaving many posts’ location as unknown. Overall, automated location inference can help compensate for the Bluesky API’s limitations, but it also introduces its own errors and blind spots that may affect the validity of geographic analyses.

This study included data from Twitter, Facebook, and Bluesky, which have a user interface or API to facilitate large-scale data collection. There are other social media platforms that can be examined, such as Reddit, YouTube, and TikTok. Furthermore, the analysis excludes private platforms (e.g., WhatsApp) and multimodal content, which may also shape public perceptions. Finally, while the observational trends underscored prevailing negative rhetoric regarding immigrants, there were limited insights regarding the impact of the rhetoric on the immigrant populations, such as impacts on health, service use, or integration. Mixed-methods research combining social-media monitoring with surveys or qualitative interviews would be beneficial in evaluating these outcomes.

## 5. Conclusion

Our decade-long, multi-platform analysis reveals that sentiment of refugee and immigrant related discourse does not follow a steady trajectory but rather is shaped by three intersectional drivers. First, policy shocks, such as new enforcement orders, prompt short-lived spikes in negativity. Second, narrative frames steer evaluations toward threat (“border crisis”) or empathy (“Dreamers,” wartime displacement). Third, platform dynamics influence which narratives are amplified. Overall, while neutral and policy-focused narratives are frequently observed, emotionally charged enforcement frames can dominate attention at critical moments. Recognizing these dynamics is essential for scholars, policymakers, and public health practitioners seeking to mitigate misinformation, reduce stigma, and foster evidence-based, compassionate migration debates in the digital public sphere. By uncovering both persistent patterns and platform-specific variations, this study offers important insights into the evolving digital discourse on immigration.

## Supplementary Material

1

## Figures and Tables

**Table 1:** Top 20 terms and the distribution of negative, neutral, and positive sentiment by platform.

	Twitter						Facebook						Bluesky			
Terms	N	Neg. (%)	Neutral (%)	Pos. (%)		Terms	N	Neg. (%)	Neutral (%)	Pos. (%)		Terms	N	Neg. (%)	Neutral (%)	Pos. (%)
Immigrants	521,881 (23.0%)	31.7	64.3	4		Immigrants	1,229,315 (20.9%)	23.4	72.1	4.4		Immigrants	185,473 (17.5%)	33.4	64	2.5
Immigration	347,287 (15.3%)	26.7	71.5	1.7		Migration	852,077 (14.5%)	17.8	78.9	3.2		Deportation	145,459 (13.7%)	43.1	55.7	1.1
Illegal	238,611 (10.5%)	37	61.1	1.8		Immigration	738,098 (12.6%)	21.3	76.9	1.8		Immigration	127,563 (12.0%)	25.3	73.3	1.3
Sanctuary city	185,539 (8.2%)	12.5	81.2	6.2		Migrants	507,668 (8.6%)	25	73.1	1.9		Migration	99,236 (9.4%)	11.5	82.3	6
ICE	182,215 (8.0%)	14.5	77.4	8		Refugees	438,814 (7.5%)	14.9	79.2	5.9		Migrants	82,481 (7.8%)	25	73.1	1.8
Migrants	102,306 (4.5%)	29.7	68	2.2		Citizenship/Naturalization	280,121 (4.8%)	20	73.3	6.4		Citizenship/Naturalization	66,490 (6.3%)	33.4	63.5	3
Refugees	91,635 (4.0%)	20.2	75.7	4.1		Deportation	245,752 (4.2%)	33.1	65.2	1.7		Asylum	55,183 (5.2%)	18.9	77.3	3.7
Illegal immigrant	85,123 (3.8%)	45.5	54	0.5		Asylum	242,214 (4.1%)	24.5	70.2	5.1		Refugees	44,955 (4.2%)	17.7	78.9	3.4
Undocumented	68,129 (3.0%)	21.6	75.2	3.2		Foreigners	190,341 (3.2%)	21.9	68.8	8.8		Sanctuary city	39,334 (3.7%)	9.7	83.4	6.9
Deportation	64,011 (2.8%)	48,4	50.4	1.1		Dreamers	133,390 (2.3%)	13	72.1	14.5		Go back where	37,606 (3.6%)	0.296	0.636	0.064
Illegal alien	58,071 (2.6%)	43.6	56	0.4		Deferred Action for Childhood Arrivals (DACA)	106,991 (1.8%)	20.8	74.2	4.9		Foreigners	28,788 (2.7%)	24.9	69.9	5.1
Border wall	47,043 (2.1%)	50	48.5	1.3		Illegal immigrant	96,304 (1.6%)	40.2	59.3	0.4		Illegal immigrant	18,492 (1.7%)	40	59.3	0.7
Border security	36,858 (1.6%)	39	60.2	0.7		Undocumented	87,759 (1.5%)	16.5	80.4	3.1		Green card	13,722 (1.3%)	16.9	71.6	11.4
Citizenship/Naturalization	30,867 (1.4%)	33.4	63.3	3.2		ICE	68,432 (1.2%)	21.2	78.1	0.6		Undocumented	12,535 (1.2%)	0.231	0.746	0.023
Illegal immigration	30,787 (1.4%)	38.9	60.7	0.4		Border patrol	53,517 (0.9%)	38.8	59.9	1.2		Illegal	11,415 (1.1%)	36.1	62.2	1.5
Asylum	27,657 (1.2%)	35.6	62.5	1.8		Illegal immigration	46,839 (0.8%)	38.4	61.2	0.4		Our country back	9,532 (0.9%)	45.9	45.8	7.5
Immigration reform	24,619 (1.1%)	18	80.5	1.5		Immigration law & policy	38,955 (0.7%)	22.2	76.6	1.2		Dreamers	9,385 (0.9%)	11.1	79.5	9.3
Immigration law & policy	21,976 (1.0%)	28	70.7	1.3		Illegal	37,070 (0.6%)	35.6	62.4	1.9		Displaced persons	9,269 (0.9%)	26.9	71.6	1.5
Build the wall	14,228 (0.6%)	41.1	57.7	1.2		Sanctuary city	36,248 (0.6%)	18.7	78.6	2.6		Border security	7,100 (0.7%)	33.2	65.9	0.9
Dreamers	13,970 (0.6%)	29.5	66.2	4.3		Temporary Protected Status (TPS)	26,329 (0.4%)	11	81	7.9		Build the wall	6,823 (0.6%)	21	75.6	3.4

## Data Availability

The datasets generated or analyzed during this study are not publicly available due to the discontinuation of the tools used to collect them. Twitter data were collected using Twitter Application Programming Interface for Academic Research (now discontinued). More information on applying for access can be found here (X, 2024). Publicly available Facebook data were collected using CrowdTangle’s user interface dashboard, a public insights tool from Meta now discontinued. More information can be found at Shiffman and Silverman (2020). Currently available tools for researchers include Meta Content Library and Content Library API (Meta, 2024). Bluesky data was collected using the platform’s publicly accessible API for searching posts, which is available to all users.

## Appendix A. Supplementary data

Supplementary data to this article can be found online at https://doi.org/10.1016/j.socscimed.2025.118715.
