# Supplementary material for: A decade of discourse: Exploring sentiments and trends around immigration on social media from 2014 to 2024
Source: Soc Sci Med. Author manuscript; Available in PMC 2026 Apr 9. (PMC13062689; doi:10.1016/j.socscimed.2025.118715)
Supplement: 1 [file NIHMS2163094-supplement-1.docx]

**Online Supplementary Materials**

**eTable 1.** Refugee and immigrant terms used in data collection

| **Keywords** |
| --- |
| aid refugee |
| alien smuggling |
| asylee |
| Asylum |
| ban islam |
| ban muslim |
| ban on muslims |
| banislam |
| banmuslim |
| banonmulsims |
| border bandit |
| border control |
| Border crisis |
| border fence |
| border hopper |
| border jumper |
| border nigger |
| border patrol |
| border security |
| border surveillance |
| border wall |
| build a wall |
| buildawall |
| buildourwall |
| buildthatwall |
| buildthedamnwall |
| buildthewall |
| buildthewallnow |
| citizenship |
| collective-expulsion |
| Deferred Action for Childhood Arrivals (DACA) |
| deport |
| deportation |
| Deportation raids |
| deported |
| deporting |
| deports |
| displaced people |
| displaced person |
| Dreamers |
| end sanctuary |
| fence hopper |
| fence-hopper |
| finishthewall |
| first generation immigrant |
| foreigner |
| foreigners |
| go back where |
| gobackwhere |
| green card |
| H-1B visa |
| help refugee |
| ICE |
| ice detention center |
| ice raids |
| ice removal |
| illegal alien |
| illegal aliens |
| illegal immigrant |
| illegal immigrants |
| Illegal immigration |
| illegalalien |
| illegalaliens |
| illegalimmigrants |
| illegals |
| imigrant |
| imigration |
| immagrant |
| immagration |
| immig |
| immigrant |
| immigrantion |
| immigrants |
| immigrates |
| immigration |
| Immigration and Customs Enforcement (ICE) |
| Immigration ban |
| Immigration detention |
| Immigration law |
| Immigration policy |
| Immigration reform |
| immigrations |
| immingrant |
| Legal immigration |
| migrant |
| migrant caravan |
| migrant protection program |
| migrants |
| migration |
| muslimban |
| Naturalization |
| naturalized |
| norefugeeban |
| openborders |
| our country back |
| ourcountryback |
| Path to citizenship |
| Public charge rule |
| rapefugee |
| refugee |
| refugeelivesmatter |
| refuges |
| repatriation |
| resettlement |
| sanctuary |
| sanctuary cities |
| sanctuary city |
| sanctuarycities |
| sanctuarycity |
| sanctuarystate |
| sanctuarystates |
| second generation immigrant |
| secure our border |
| smuggling aliens |
| Smuggling immigrants |
| syrianrefugee |
| Temporary protected status |
| Temporary Protected Status (TPS) |
| Title 42 |
| travel ban |
| Unauthorized immigrant |
| undocumented |
| UNHCR |
| we welcome refugee |
| welcome refugee |
| welcomerefugee |
| wetback |
| wetbacks |
| work permit |

eFigure 1 presents the average sentiment scores toward both immigrants and refugees combined from 2014 to 2023. Overall, sentiment remained predominantly negative nationwide, with no state exhibiting a neutral or positive average score during the observed period. Several states exhibited notably negative sentiment, as indicated by darker shades on the map and lower sentiment scores. These states include Montana (-0.335), Wyoming (-0.341), Oregon (-0.327), and Nevada (-0.334) in the West. Oklahoma (-0.353), Arkansas (-0.331), Mississippi (-0.333), Alabama (-0.339), Kentucky (-0.353)and Louisiana (-0.332) in the South. , Florida (-0.329), South Carolina (-0.340), and Delaware (-0.360) in the South Atlantic. Among these, West Virginia stands out as the most negative state overall, with a sentiment score of -0.371. Northeastern and Midwestern states such as New Jersey (-0.327) and Michigan (-0.328) also exhibited notably negative sentiment.. In contrast, only a few regions reflected relatively less negative sentiment, shown as lighter tones on the map. Washington, D.C., although not visible due to its small geographic footprint, recorded the least negative score at -0.20, suggesting a comparatively more neutral discourse. Similarly, Massachusetts (-0.252), New York (-0.260), and North Dakota (-0.272) exhibited the most moderate sentiment among states. These regions, primarily located in the Northeast and upper Midwest suggest less negative public narratives surrounding immigration and refugee issues. Hawaii, Alaska, and Puerto Rico were not included in the map visualizations due to geographic constraints but are important to note in the broader analysis. Their sentiment scores align with the overall national trend of predominantly negative sentiment toward immigrants and refugees. Hawaii recorded a sentiment score of -0.313, Alaska had a score of -0.331 and Puerto Rico, a significant U.S. territory, recorded a sentiment score of -0.290. These values contribute to a more complete understanding of public sentiment across all U.S. regions during the 2014 - 2023 period.

**eFigure 1.** Average sentiment for all immigrant and refugee keywords by state (2014 - 2023)


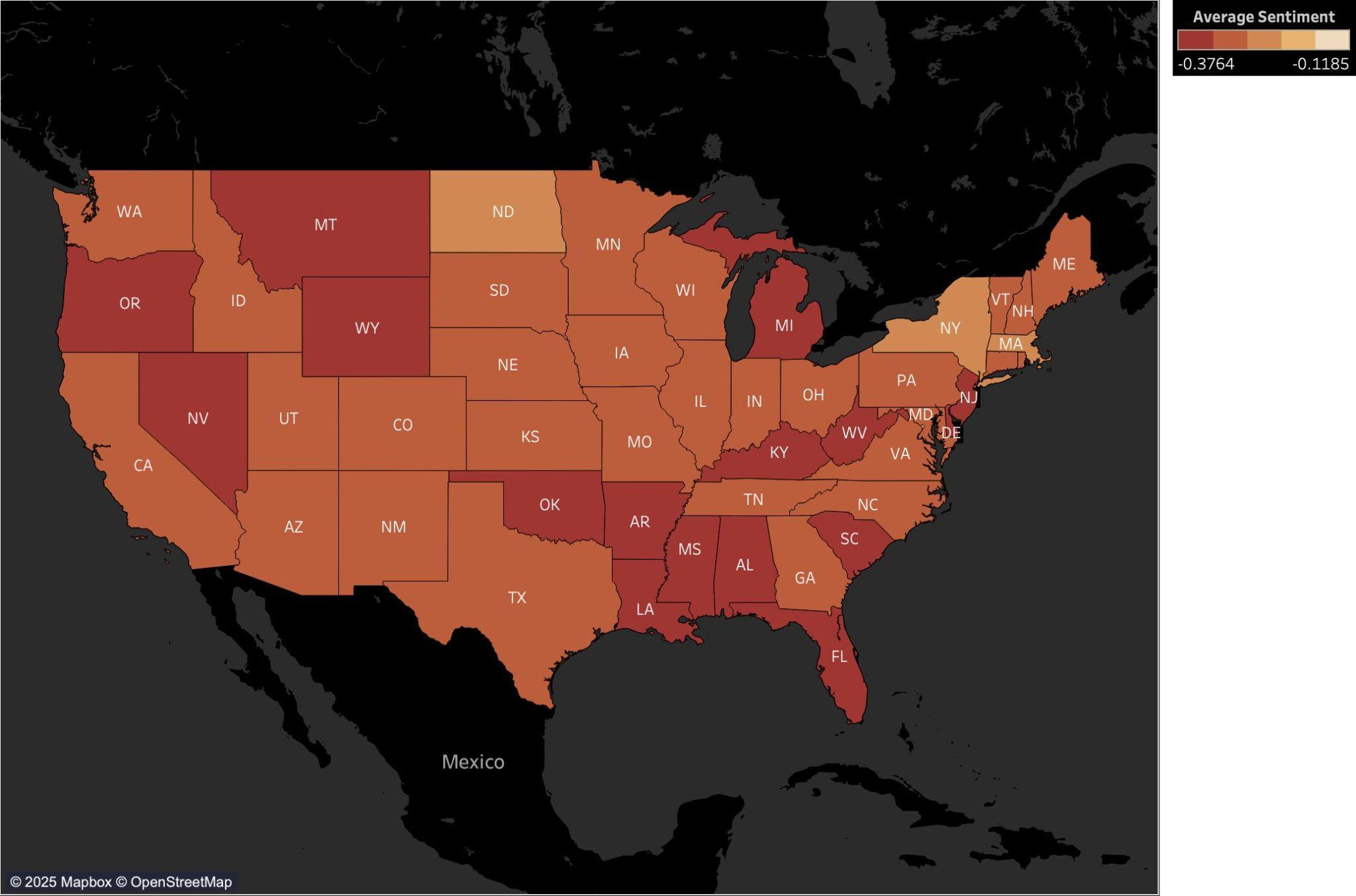


**eTable 2.** State-level count and average sentiment for all immigrant and refugee keywords, 2014-2023

| **Midwest** | **Northeast** | **South** | **West** | **Territories** |
| --- | --- | --- | --- | --- |

| State | Frequency | Average Sentiment |
| --- | --- | --- |
| MI | 37262 | -0.328 |
| IN | 24806 | -0.323 |
| SD | 2224 | -0.323 |
| OH | 55924 | -0.315 |
| KS | 12032 | -0.314 |
| WI | 17877 | -0.31 |
| MO | 25508 | -0.304 |
| NE | 6775 | -0.295 |
| IA | 10809 | -0.293 |
| MN | 23132 | -0.286 |
| IL | 63563 | -0.281 |
| ND | 1476 | -0.272 |
| NJ | 57379 | -0.327 |
| PA | 76508 | -0.326 |
| CT | 19766 | -0.32 |
| ME | 6688 | -0.312 |
| VT | 5372 | -0.309 |
| NH | 7502 | -0.308 |
| RI | 5779 | -0.294 |
| NY | 170518 | -0.26 |
| MA | 54091 | -0.252 |
| WV | 6763 | -0.371 |
| DE | 5753 | -0.36 |
| OK | 15377 | -0.353 |
| KY | 16397 | -0.353 |
| SC | 24828 | -0.34 |
| AL | 21352 | -0.339 |
| MS | 8852 | -0.333 |
| LA | 19642 | -0.332 |
| AR | 11680 | -0.331 |
| FL | 152001 | -0.329 |
| NC | 46755 | -0.319 |
| GA | 52649 | -0.317 |
| TN | 31006 | -0.315 |
| VA | 52639 | -0.313 |
| TX | 191714 | -0.299 |
| MD | 42320 | -0.294 |
| WY | 1713 | -0.341 |
| MT | 3840 | -0.335 |
| NV | 30442 | -0.334 |
| AK | 4330 | -0.331 |
| OR | 25158 | -0.327 |
| NM | 11166 | -0.32 |
| AZ | 52384 | -0.316 |
| HI | 7717 | -0.313 |
| ID | 6924 | -0.311 |
| CO | 28762 | -0.307 |
| WA | 43167 | -0.3 |
| UT | 10814 | -0.298 |
| CA | 281820 | -0.296 |
| GU | 82 | -0.427 |
| PR | 1848 | -0.29 |
| VI | 147 | -0.279 |
| DC | 46275 | -0.20 |
